# Quantitative Phase and Intensity Microscopy Using Snapshot White Light Wavefront Sensing

**DOI:** 10.1038/s41598-019-50264-3

**Published:** 2019-09-24

**Authors:** Congli Wang, Qiang Fu, Xiong Dun, Wolfgang Heidrich

**Affiliations:** Visual Computing Center, King Abdullah University of Science and Technology, Thuwal, 23955-6900 Saudi Arabia

**Keywords:** Imaging and sensing, Phase-contrast microscopy

## Abstract

Phase imaging techniques are an invaluable tool in microscopy for quickly examining thin transparent specimens. Existing methods are limited to either simple and inexpensive methods that produce only qualitative phase information (e.g. phase contrast microscopy, DIC), or significantly more elaborate and expensive quantitative methods. Here we demonstrate a low-cost, easy to implement microscopy setup for quantitative imaging of phase and bright field amplitude using collimated white light illumination.

## Introduction

Due to negligible absorption in the visible spectrum, most living cells exhibit low contrast under bright field microscopy, which prevents detailed examinations. In comparison, phase imaging detects minute changes in phase when light propagates through the cell morphology, and has become the prevalent approach for fine cell strucfture distinction without employing higher radiation powers.

Two classical methods for phase imaging are phase-contrast microscopy^1^ and differential interference contrast (DIC) microscopy^2^. These methods utilize additional simple imaging modules (e.g. annulus rings or Nomarski prisms) to convert the phase shifts into brightness changes. However, the conversion is not linear and the recorded image on the detector only indicates qualitative pseudo phase information, and is often substantially different from the real phase shift.

Many quantitative phase imaging techniques have been proposed^3^. One notable technique is the defocused-based phase imaging^4–8^ based on Transport of Intensity Equation (TIE)^9^, where two or more intensity images are recorded at several closely spaced planes (usually 10 μm to 100 μm apart). From these images the phase shifts are numerically reconstructed, for example by sequentially solving two Poisson equations^9^. However obtaining the defocused images, requires delicate experimental setups, e.g. well-aligned mechanical scanning or specifically-designed wavefront-separation components, hence preventing easy lab implementations. Digital holographic microscopy^10,11^ records multiple interferograms under different reference beams (via phase shifts or frequency shifts), then post-processing via Fourier analysis^12^ interprets the interferograms to recover the sample intensity and phase, but suffers from the challenging ill-posed 2D phase unwrapping problem for fringe pattern analysis. Variants of this technique include off-axis digital holography^13^, *τ* interferometers^14,15^, Lloyd’s mirror^16^, and many others. Similar to the TIE-based phase imaging technique, digital holography requires additional optical components to realize the different reference beams. One special variant is spatial light interference microscopy^17^, which minimizes optical path light coherent sensitivity. Other interference-based methods include diffraction phase microscopy^18^, similar to Mach-Zehnder interferometry, but overlays the reference and phase-shift beams in the same optical path, by preserving the 1^st^ and 0^th^ order diffraction from a grating using a customized aperture at Fourier plane. Dynamic interference microscopy^19^ employs a micro-polarizer array and phase-shifts are encoded into polarization intensity change. Apart from above deterministic phase methods (i.e. closed-form formulas for phase-shifts), undetermined or iterative phase solving methods are emerging that rely on phase-retrieval algorithms, for example coded aperture phase imaging^20,21^, which employs a random aperture for phase encoding and an inverse problem is solved via a customized phase retrieval algorithm, or the structured light illumination techniques^22,23^ that capture diffraction holograms under different background illuminations for subsequent numerical phase-retrieval. Ptychography^24,25^ and Fourier ptychographic microscopy^26,27^ shows the great potential for super-resolution intensity and phase measurement beyond diffraction-limit via multiple angle illumination and a joint phase-retrieval algorithm. Last, the near-field speckle pattern X-ray imaging^28–30^ that utilizes the phase-stepping method is able to obtain phase and scattering field measurements via numerical deconvolution^31,32^. More recently programmable wavefront sensing techniques have been proposed using programmable spatial light modulators^33^.

These approaches measure the actual optical path length differences in the specimen and convert them into thickness, enabling quantitative visualization of sample optical density via the measured phase shift, and some even offer super-resolution or even dark field image reconstructions. However, these approaches require specialized, expensive and complicated setups, coherent illumination, or a long acquisition time prohibited for real time applications, and the flexibility is lost to quickly obtain normal irradiance images and phase images on an ordinary commercially available microscope.

In this work we demonstrate quantitative phase and intensity imaging based on improvements of our previous work on high-resolution wavefront sensing based on speckle-pattern tracking^34^. Our method only requires minor modifications to a conventional microscope and works under white light illumination. Wavefront sensing using speckle tracking technique was first proposed in X-ray phase imaging^32,35–38^, and then for optical wavefront retrieval^34,39^, adaptive optics^40^, and trial lens metrology^41^. Simultaneous reconstruction for absorption, phase, and dark field images from one single speckle-pattern measurement image have also been shown^42–44^. This speckle tracking technique can be regarded as a generalization of Shack-Hartmann^45^ or Hartmann masks^46^. Closely related special cases are the shearing interferometers^47,48^ and variants^49–53^ that enable closed-form solutions in Fourier domain for wavefront retrieval. See Supplementary Information for further discussions.

We improve our previous work^34^ by introducing a new, non-linear model that is a generalization of TIE, which is able to work with temporally incoherent light while being more robust to alignment and less sensitive to noise. Derivation details and analysis of this model are provided in the Supplementary Information. Using this model combined with modern numerical optimization frameworks^54^, customized algorithms are proposed for simultaneous recovery of amplitude and phase. Superiority of the proposed over classical speckle-pattern tracking algorithms is verified using both synthetic and laboratory data. We analyze optical diffraction for speckle-pattern tracking techniques in wave optics and unify previous models in the Supplementary Information, but also offer in this main document a simple yet equivalent ray optics approach revived from previous computational caustic lens design research^55^.

## Results

### Sensor principle

**Figure 1:**
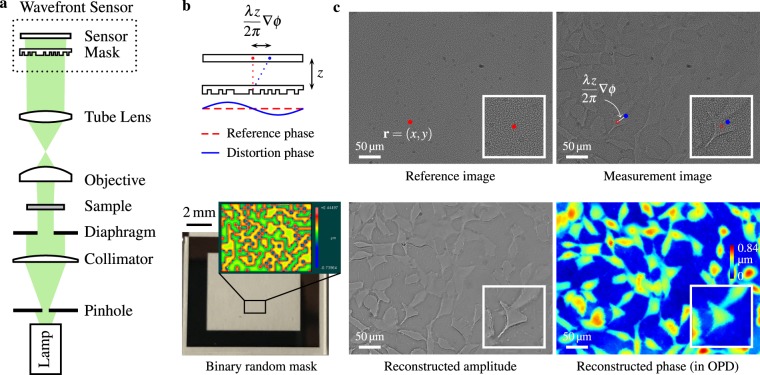
Quantitative phase imaging with a coded wavefront sensor. (**a**) Optical setup for our prototype quantitative phase microscope. Intensity sensor and sample are at conjugate planes, collimated white light is configured for sample illumination. **(b)** Principle of the coded wavefront sensor. A normal intensity sensor is overlaid by a binary phase mask whose Zygo interference map is shown as inset image. **(c)** The diffraction pattern moves in proportion to local wavefront slopes, as indicated by the red and blue dots. Raw captured images reveal a diffraction pattern movement caused by distortion phase. Inset images are magnified close-ups for regions of interest. Given the image pair, intensity and phase images can be altogether numerically reconstructed for unstained thin transparent cells. The sample contains HeLa cells taken under a ×20 Mitutoyo plan apochromat objective, 0.42 NA.

The setup for the proposed quantitative phase imaging microscope requires two modifications to a conventional digital research microscope: (1) replacing the camera with a high resolution coded wavefront sensor^34^, and (2) modifying the trans-illumination module for collimated but temporally incoherent light, i.e. broadband spectrum illumination. Figure 1(a) shows the optical setup, as well as the coded wavefront sensor, which consists of a random phase mask and a normal intensity sensor. The phase mask is placed close to the sensor at a distance of *z* ≈ 1.5 mm. See Methods for *z* distance calibration details.

Under collimated illumination (thus spatially coherent), the observed *reference image I*_0_(**r**) is a diffraction pattern of the high frequency mask, as shown in Fig. 1(b). When a sample is introduced into the optical path, the wavefront is distorted, and the diffraction pattern changes accordingly. Crucially, the wavefront impinging on the sensor is encoded into the movement of the speckle pattern in a *measurement image I*(**r**). We have previously shown^34^ that the wavefront slopes ∇*ϕ* is optically encoded in image displacements, also known as the “optical flow” in computer vision, written as:

1 $$I({\bf{r}})={I}_{0}({\bf{r}}-\frac{\lambda z}{2\pi }\nabla \varphi ),$$

where *z* is the distance between mask and sensor, *λ* is wavelength, and ▽ is the gradient operator. See Methods and Supplementary Information for a physical optics derivation of this relationship. When dispersion is negligible, this coded wavefront sensor works well under temporally incoherent broadband illumination, and hence the retrieved wavefront can be directly mapped to optical path differences (OPD, defined as ${\rm{O}}{\rm{P}}{\rm{D}}=\frac{\lambda }{2\pi }\varphi$), in the sense that the refractive index *n* is constant with respect to different wavelengths (weak dispersion assumption) and hence OPD = (*n* − 1) × *d* is only a variable of sample thickness *d*. As in other white light wavefront sensing techniques such as Shack-Hartmann, a nominal wavelength (e.g. 532.8 nm) is needed for conversion between OPD and wavefront/phase. The reference image *I*_0_(**r**) only needs to be captured once prior to any sample measurements, thus this method enables snapshot phase measurement at video rates.

### Simultaneous intensity and phase reconstruction

While the observed measurement image *I*(**r**) is modulated with the speckle pattern, this pattern can be computationally removed to recover an intensity image free from speckle. To this end, we re-visit the underlining principle of Wang *et al*.^34^. Given that the biological sample is weak in absorption, resulting in a relatively flat intensity profile, simultaneous amplitude and phase estimation can be achieved by modifying the original data term with additional considerations on sample amplitude and diffraction. We generalize Eq. (1) and previous speckle-pattern tracking models^34,37,39,44,56^, via our analysis, as:

2 $$I({\bf{r}}+\frac{\lambda z}{2\pi }\nabla \varphi )=\mathop{\underbrace{{|A({\bf{r}})|}^{2}(1-\frac{\lambda z}{2\pi }{\nabla }^{2}\varphi )}}\limits_{|\tilde{A}{|}^{2}}{I}_{0}({\bf{r}}),$$

where *A*(**r**) is the unknown sample amplitude that we would also like to recover. See Eq. (10) in Methods for a short derivation from ray optics, and the relationship between TIE and Eq. (2). We refer to the Supplementary Information a full physical optics derivation that accounts for sample diffraction. Given a reference image *I*_0_(**r**) and measurement image *I*(**r**), we simultaneously recover intensity |*A*(**r**)|^2^ and phase *ϕ*(**r**) from Eq. (2). This is a numerically difficult task, that can be made more robust by incorporating prior information on the phase and on the intensity, respectively denoted as Γ_phase_(*ϕ*) and ${\Gamma }_{{\rm{intensity}}}(|\tilde{A}{|}^{2})$. The phase and intensity reconstruction process can then be phrased as an optimization problem:

3 $$\mathop{{\rm{minimize}}}\limits_{\tilde{A},\,\varphi }\,{\Vert I({\bf{r}}+\frac{\lambda z}{2\pi }\nabla \varphi )-|\tilde{A}{|}^{2}{I}_{0}({\bf{r}})\Vert }_{2}^{2}+{\Gamma }_{{\rm{phase}}}(\varphi )+{\Gamma }_{{\rm{intensity}}}(|\tilde{A}{|}^{2}),$$

where Γ_phase_(*ϕ*) and ${\Gamma }_{{\rm{intensity}}}(|\tilde{A}{|}^{2})$ represent terms for gradient and Laplacian sparsity and smoothness, for phase and irradiance respectively (see Methods for details). We optimize each unknown term in an alternating fashion (see Supplementary Information for details). This process converges quickly in a few (≈3) alternating steps. After obtaining $\tilde{A}$ and *ϕ*, pure sample amplitude can afterwards be computed by subtracting intensity changes from refocusing (i.e. caustics) due to local wavefront curvature, which, however is a small effect (*λz*|*∇*2*ϕ*| ≪ 2π):

4 $$A=\tilde{A}\sqrt{1+\frac{\lambda z}{2\pi }{\nabla }^{2}\varphi }.$$

Thanks to modern optimization schemes, the algorithm can be efficiently parallel implemented, enabling GPU acceleration (see Methods for solver performance). Our method improves prior speckle-pattern tracking techniques both by accounting for local wavefront curvature (the caustic effect) and amplitude in the model which is crucial for absorption-refraction tangled scenarios in typical microscopy imaging, and by jointly estimating *A* and *ϕ* directly from the raw speckle data. Previous approaches, on the other hand, either fail to consider amplitude^34,37,39^ and the local curvature term, or apply sequential calculations for estimating *A*, ∇*ϕ*, and *ϕ* in separate stages^37,39,42,44^, which limits the total reconstruction performance.

### Characterization of a microlens array

**Figure 2:**
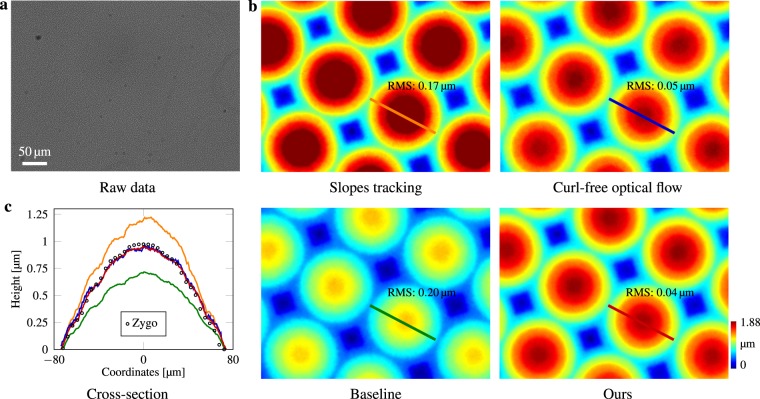
Accuracy validation measurement using a microlens array. Image was taken under a ×20 Mitutoyo plan apochromat objective, 0.42 NA. **(a)** Raw data. **(b)** In comparison with the manufacturer’s specification, both curl-free optical flow^34^ and our proposed algorithm estimate the height with high accuracy. By comparison, classical slope tracking^37,39^ overestimates, and the baseline method^44^ underestimates the phase shifts. **(c)** For cross-section comparison all heights have been normalized to start from 0 μm.

To demonstrate the accuracy of our quantitative phase imaging microscope, a square grid microlens array (MLA150-7AR-M, Thorlabs) was imaged, for which each lenslet is of 150 μm apart and 6.7 mm back focal length. We compare our method to both Zygo measurements and a classical baseline speckle-pattern tracking algorithm^44^ in Fig. 2. The measured optical path differences are converted to physical thickness using a refractive index of 1.46 at 532.8 nm (fused silica). Figure 2(c) shows cross-sectional thickness profiles for one of the microlenses. Our reconstructed height matches Zygo measured data, which is also indicated by the root-mean-square (RMS) error computed for each cross-section microlens phase profile, whereas the baseline algorithm is 0.20 μm. This laboratory result validates that, for visible light optical microscopy phase imaging, our proposed numerical algorithm outperforms classical speckle-tracking algorithms, which suffer from phase reconstruction error because of their sequential nature. Our previous algorithm curl-free optical flow^34^ is overall in good agreement with both the Zygo measurements and our proposed method, however there exists high-frequency noise. See Supplementary Information for implementation details.

### Influence of irradiance-varying samples

**Figure 3:**
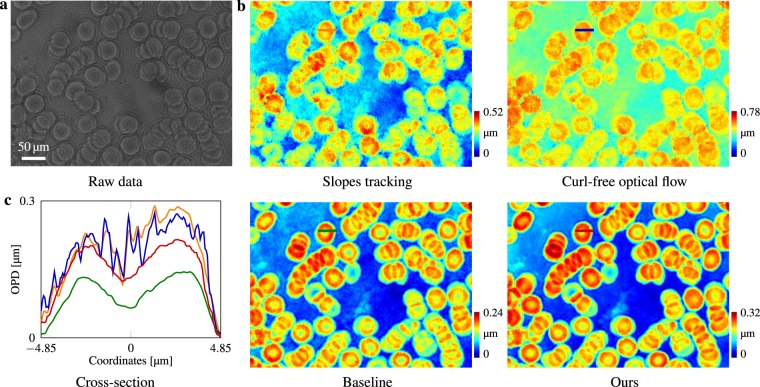
Phase reconstruction comparison for an air-dried human red blood cell smear using different methods. Image was taken under a ×100 Mitutoyo plan apochromat objective, 0.70 NA. **(a)** Raw data. **(b)** Reconstruction phase shifts from different methods. **(c)** Cross-section profile of a single cell, phases are normalized to start from height 0. The two pure-tracking methods based on Eq. (1), i.e. slopes tracking^37,39^ and curl-free optical flow^34^, are vulnerable to amplitude changes and fail to reconstruct the bowl-like indentations because their model neglects sample amplitude. Though based on Eq. (2), traditional baseline method^44^ underestimates the phase shifts (as well in Fig. 2), whereas our reconstruction height maps match the metrology statistics^68^.

Figure 3 demonstrates the advantage of Eq. (2) over Eq. (1) on an air-dried human blood cell smear. Phase-only reconstruction results are shown to compare different methods. In such an irradiance-varying situation, previous pure flow-tracking algorithms^34,37,39^ (based on Eq. (1)) are vulnerable to the amplitude changes. Classical baseline methods for speckle-pattern tracking^44^ based on local window intensity estimation and windowed correlation, though based on Eq. (2), however, tend to underestimate the phase shifts, as also shown previously in the validation experiment Fig. 2. Further results and more synthetic numerical comparisons with the baseline method can be found in Supplementary Information.

### Imaging of transparent cells

**Figure 4:**
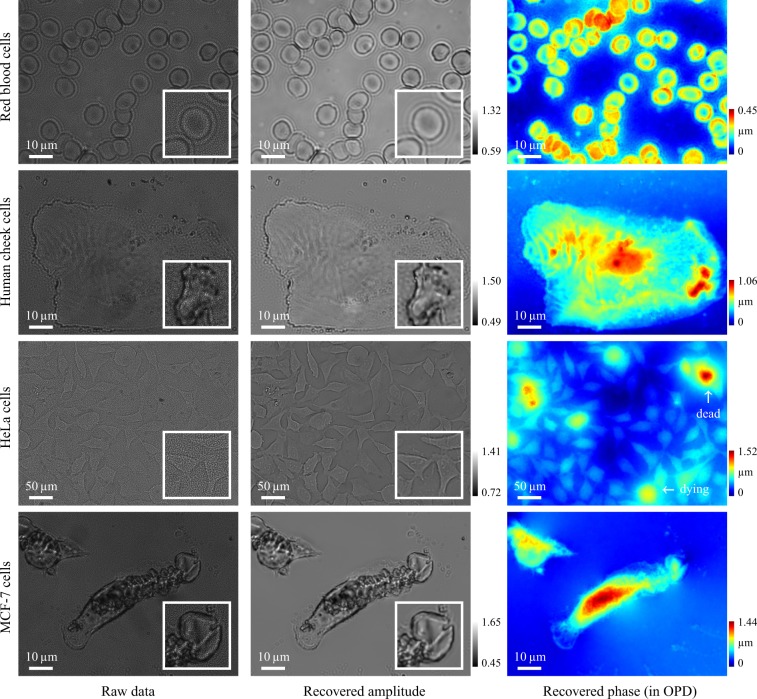
Experimental results with unstained thin transparent cells with the proposed quantitative phase imaging pipeline. Images were taken under ×20 (0.42 NA) and ×100 (0.70 NA) Mitutoyo plan apochromat objectives. Phase images are shown in terms of OPD, where optically thick structures reveal informative details regarding the samples. Inset close-up images (normalized for better visualization) show local areas of interest, note in the recovered amplitude images the speckle patterns have been fully removed. Cell live/dead viability can be quickly examined via phase measurements.

We also show the capability of imaging unstained thin transparent cells using the proposed quantitative phase microscopy. From one single raw speckle data, simultaneous amplitude and phase images are numerically reconstructed as shown in Fig. 4 for different cells. The phase images are shown as the measured OPD, and the actual height of the samples can be calculated when true refractive indexes are known. Noticeably, the torus structures of the red blood cells have been plausibly reconstructed. For the human cheek cells sample, the phase map indicates its biological structure with height informational details (compared to bright field imaging). For the HeLa cells sample, the humongous phase changes of the dying cells reveal the bio-activity, providing informative contrast details beyond original bright field microscopy or even qualitative phase microscopy methods e.g. phase-contrast or DIC. For the MCF-7 cells, note how our method enables fine phase reconstruction at the boundaries while preserving the original bright field image. Since quantitative phase information is obtained, all other phase microscopy such as phase-contrast and DIC can be numerically simulated. More experimental results are in Supplementary Information.

### Digital refocusing

**Figure 5:**
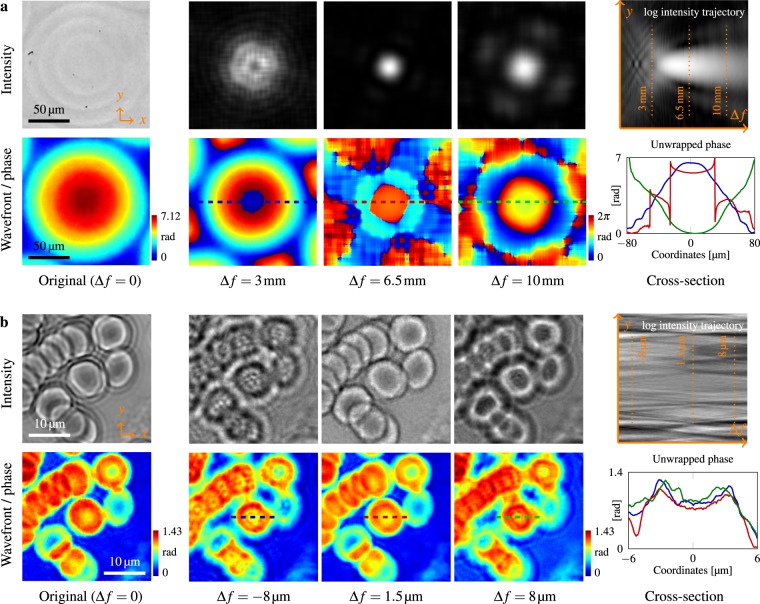
Post-capture refocusing by digitally propagating defocus distance Δ*f* with the acquired complex field. (**a**) Digital refocusing of a microlens scalar field in Fig. 2 is made possible once its intensity and wavefront are obtained via our approach. For different Δ*f*, the defocusing evolution of a diffraction-limited spot can be emulated. **(b)** Digital refocusing can also be performed to remove the original ringing artifacts due to defocusing. Refocusing blood cells in Fig. 4 sharpens the intensity images, and provides a more plausible phase profile for originally out-of-focus samples.

Finally, we demonstrate the digital refocusing capability of the proposed technique. Since the full complex field is acquired, similar to digital holography, we are able to perform digital refocusing on the recovered intensity and wavefront. However unlike digital holography, our approach employs broadband illumination (multiple wavelengths), and the concept of phase is ill-defined. Hence, we define a nominal wavelength *λ* = 532.8 nm, and convert the obtained wavefront (OPD-based [μm]) into phase (unitless [rad]). Two examples are shown in Fig. 5. In Fig. 5(a), the previously obtained microlens is digitally propagated through different defocus distance Δ*f*. The best focus distance matches the back focal length provided by manufacturer. Cross-section phase profiles also demonstrate evolution of the propagating wavefront, from converging to almost flat, and finally to diverging. In Fig. 5(b), digital refocusing of blood cells to the correct focus plane sharpens the edges of the originally blurry intensity image, and the bowl-like indentation is more obvious and plausible for the central cell, as shown in the cross-section.

## Discussion

All data required to determine the phase shift are gathered in a single snapshot utilizing a coded wavefront sensor^34^, so there is no need for scanning, which however is one potential future research direction to obtain scattering images^44,57,58^. Specific grating (mask) designs or multi-layer designs^59,60^ are potential directions. Fast simultaneous amplitude and phase acquiring advances current tomography techniques^61–63^ beyond X-ray. Short exposure times freeze motion, allowing a capture for fast movements. From the reconstructed phase, different types of phase imaging techniques such as phase contrast and DIC images are also obtained simultaneously along with the recovered optical thickness.

The proposed technique can be further extended to higher magnifications, immersion objectives, higher numerical apertures, to measure thin and transparent specimens under white light illumination. The avoidance of laser illumination and the self-reference characteristic of our sensor offer a non-destructive means of observing and quantifying biological behavior and cellular dynamics over time, without suffering from environmental vibration noise, and at a harmless lighting level.

Despite these advantages, there are limitations for our technique: First, our technique requires a spatially coherent illumination (though temporally incoherent), thus a collimated illumination. For sufficient sensor pixel sampling rate, the spatial resolution is limited to the objective NA. Because of the spatially coherent illumination requirement, on the same microscope, the spatial resolution of our recovered bright field image, in theory, is at worst two times less than the usual bright field microscopy setting under Köhler incoherent illumination^64^. Second, our sensor relies on speckle-pattern tracking for wavefront recovery. That said, our sensor is in essential a slope wavefront sensor such as Shack-Hartmann^65^. According to Eq. (2), the caustic effect $(\frac{\lambda z}{2\pi }{\nabla }^{2}\varphi )$ and sample intensity (|*A*|^2^) are coupled. Consequently, for highly curved wavefronts *ϕ*, the residual wavefront recovery error creates a caustic effect, which modulates the recovered bright field image, causing ambiguous interpretation of the recovered bright field image, as a result of a mixture of caustic effect and sample intensity. See also Methods and Supplementary Information for discussions.

We have demonstrated the proposed quantitative phase imaging pipeline for simultaneous amplitude and phase reconstruction via minor modifications on an ordinary optical microscopy. Our new theoretical model establishes the connection between speckle-pattern tracking and TIE-based determined phase retrieval. Powered by an efficient joint optimization numerical scheme, we show computational potentials for better performance using the same raw speckle image. Through imaging different transparent cells, amplitude and phase reconstruction results are present. We believe using the coded wavefront sensor, without additional hardware, the potential to transform an ordinary bright field microscopy to multi-functional microscopy for simultaneous quantitative phase and amplitude imaging opens up new research directions and inspiring applications.

## Methods

### Theory

#### Ray optics derivation

**Figure 6:**
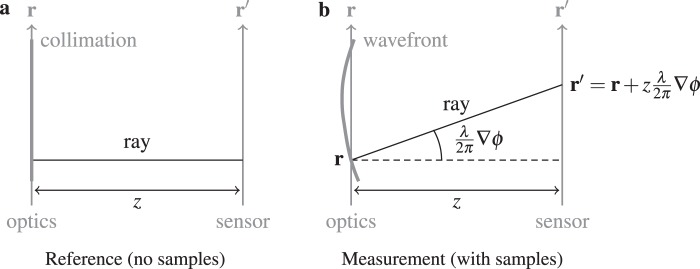
Ray optics derivation, the geometry model for referring Eq. (2). (**a)** Reference image *I*_0_ is recorded only once prior to any sample measurements. **(b)** When there are samples, snapshot measurement image *I* is recorded.

Here we replicate derivation from Damberg and Heidrich^55^ and modify it for referencing Eq. (2). Equivalent but precise wave optics derivations can be found in Supplementary Information. Let **r** and **r**′ be the coordinates at the mask plane and sensor plane respectively, as in Fig. 6, for one single ray, by geometry at wavelength *λ*:

5 $${\bf{r}}{\boldsymbol{^{\prime} }}={\bf{r}}+z\frac{\lambda }{2\pi }\nabla \varphi ({\bf{r}}),$$

where we employ small angle approximation that sin*θ* ≈ *θ* when $\frac{\lambda }{2\pi }\nabla \varphi ({\bf{r}})=\theta \ll 1$. Since for each local differential area the irradiance energy is conserved, therefore there is a simple relationship between the intensity *I*(**r**′) on the sensor plane and the intensity *J*(**r**) at mask plane:

6 $$I({\bf{r}}{\boldsymbol{^{\prime} }})\,{\rm{d}}{\bf{r}}{\boldsymbol{^{\prime} }}=J({\bf{r}})\,{\rm{d}}{\bf{r}}.$$

Differentiate Eq. (5), we get:

7 $${\rm{d}}{\bf{r}}{\boldsymbol{^{\prime} }}=(1+\frac{\lambda z}{2\pi }{\nabla }^{2}\varphi ({\bf{r}})){\rm{d}}{\bf{r}}.$$

Given Eq. (7), Eq. (6) can be reformulated as:

8 $$I({\bf{r}}{\boldsymbol{^{\prime} }})=\frac{1}{(1+\frac{\lambda z}{2\pi }{\nabla }^{2}\varphi ({\bf{r}}))}J({\bf{r}})\approx (1-\frac{\lambda z}{2\pi }{\nabla }^{2}\varphi ({\bf{r}}))J({\bf{r}}),$$

where the approximation is valid because $\frac{\lambda z}{2\pi }|{\nabla }^{2}\varphi ({\bf{r}})|\ll 1$. By Eq. (5), finally we arrive at:

9 $$I({\bf{r}}+\frac{\lambda z}{2\pi }\nabla \varphi )=(1-\frac{\lambda z}{2\pi }{\nabla }^{2}\varphi )J({\bf{r}}).$$

In our case *J*(**r**) is the diffraction pattern image of the modulation mask. Under collimated illumination we obtain the reference image *I*_0_(**r**) = *J*(**r**). When imaging at scalar field *A*(**r**)exp[j*ϕ*(**r**)], Eq. (9) can be formulated (i.e. as Eq. (2)):

10 $$I({\bf{r}}+\frac{\lambda z}{2\pi }\nabla \varphi )=|A({\bf{r}}){|}^{2}(1-\frac{\lambda z}{2\pi }{\nabla }^{2}\varphi ){I}_{0}({\bf{r}}).$$

#### Wavefront resolution analysis

Above derivation for Eq. (2) requires small curvature assumption that $\frac{\lambda }{2\pi }|{{\rm{\nabla }}}^{2}\varphi ({\bf{r}})|=|{{\rm{\nabla }}}^{2}{\rm{O}}{\rm{P}}{\rm{D}}|\ll 1/z$. This condition determines the wavefront resolution of our technique: the incoming wavefront local curvature must be small enough, indicated by upper bound 1/*z*. This upper bound could be interpret in terms of Fourier harmonics, to derive the phase transfer function for our sensor. Let OPD = *H*cos*ωx*, then:

11 $$|{\nabla }^{2}{\rm{OPD}}|\ll 1/z\Rightarrow H{\omega }^{2}|\,\cos \,\omega |\le H{\omega }^{2}\ll 1/z\Rightarrow H\ll {H}_{{\rm{upper}}\_{\rm{bound}}}=\frac{1}{z{\omega }^{2}}.$$

**Figure 7:**
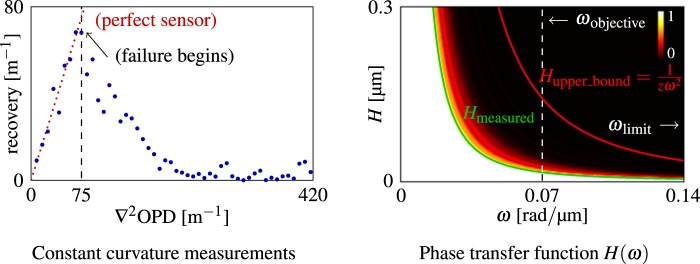
Wavefront resolution analysis and the phase transfer function. Our recovered wavefront curvatures are plotted for groups of gradually increasing constant phase curvature ▽^2^OPD. For ▽^2^OPD > 75 m^−1^, our sensor begins to fail for recovery. Based on this measured failure starting curvature, we are able to compute the valid area (valued from 0 to 1) for phase transfer function based on the recovery error (larger the error, smaller the value). According to Eq. (11), our measured tight bound *H*_measured_ and the upper bound *H*_upper_bound_ are shown. However our prototype resolution is limited by the optical resolution of the microscope objective *ω*_objective_ = 0.07 rad/μm instead of the measured limit *ω*_limit_ = 0.14 rad/μm.

**Figure 8:**
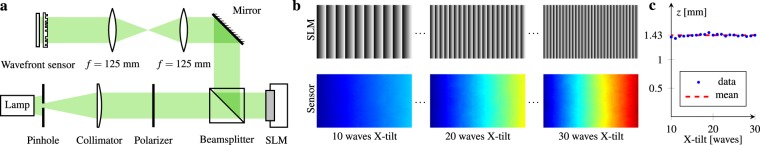
Calibration of wavefront sensor scaling factor. (**a**) Optical setup for wavefront sensor calibration. The sensor plane and the SLM plane are in conjugate. **(b)** Input grayscale phase images to the SLM and reconstructed wavefront surfaces (after image resizing). **(c)** Estimated mask-to-sensor distances from **(b)** over different ground-truth slopes, and the mean.

However, this theoretical upper bound 1/*z* is not tight, and the actual performance needs to be measured experimentally. Results are shown in Fig. 7, where we measured groups of gradually increasing curvature phase maps using the setup in Fig. 8(a). We notice our sensor starts to fail at wavefront curvature of 75 m^−1^, whereas the upper bound indicates 1/*z* ≈ 700 m^−1^. It agrees with the general rule of thumb that ≪ indicates an order of magnitude relationship. Given this number, we are able to compute the phase transfer function *H*_measured_(*ω*), i.e. the practical wavefront resolution.

However, the actual resolution also depends on the microscopy objective, since current image sensor technology makes it easy to choose sensor resolutions that exceed the optical resolution of the microscope, especially in high magnification microscopy. Most of our experiments were conduct with a ×100 objective (0.70 NA), at nominal wavelength *λ* = 532.8 nm, corresponding to Rayleigh resolution of 100 × 0.61*λ*/NA = 46.4 μm (i.e. *ω*_objective_ = 0.07 rad/μm), which is 7.2 times larger than our prototype sensor pixel size 6.45 μm (i.e. *ω*_pixel_ = 0.49 rad/μm). Given the measured limit *ω*_limit_ = 0.14 rad/μm in Fig. 7, we have *ω*_objective_ < *ω*_limit_ < *ω*_pixel_, hence the wavefront resolution is limited by *ω*_objective_, i.e. the full system is limited by the optical performance of the microscope objective. Note that *ω*_objective_ could be improved by using objectives with higher NA, and *ω*_limit_ could also be improved by adjusting the distance *z* between mask and sensor, to which the theoretical upper bound is inversely proportional. This provides a rich design space for performance optimized systems based on our approach.

#### Connection to transport-of-intensity equation

The Transport-of-Intensity Equation (TIE), a.k.a. the irradiance transport equation^9,66^, can be derived from Eq. (10) by performing a first-order Taylor approximation for $I({\bf{r}}+\frac{\lambda z}{2\pi }\nabla \varphi )$ around **r**, and re-arranging the terms:

12 $$\nabla I({\bf{r}})\cdot \nabla \varphi +|A({\bf{r}}){|}^{2}{I}_{0}({\bf{r}}){\nabla }^{2}\varphi =\frac{k}{z}(|A({\bf{r}}){|}^{2}{I}_{0}({\bf{r}})-I({\bf{r}})).$$

In traditional TIE setups, there is no masks, and hence *I*_0_(**r**) = 1. To see it more clearly, let the image captured at the original mask plane be *I*_1_(**r**) = |*A*(**r**)|^2^, and the second image captured at the original sensor plane be *I*_2_(**r**) = *I*(**r**), then Eq. (12) can be reformulated as:

13 $$\nabla {I}_{2}\cdot \nabla \varphi +{I}_{1}{\nabla }^{2}\varphi =\frac{k}{z}({I}_{1}-{I}_{2})\approx -\,k\frac{\partial \bar{I}}{\partial z}.$$

Let *I*_1_(**r**) ≈ *I*_2_(**r**) ≈ $\bar{I}({\bf{r}})$ we arrive at the standard form of TIE, when *z* → 0. Further discussions are found in the Supplementary Information.

### Computation

Compared to the general wavefront sensing situations where the target phase is smooth, microscopy phase images contain more details and many sharp edges. To better formulate and regularize accordingly, we incorporate additional gradient and Hessian priors into the original problem^34^ to regularize Eq. (2). Introducing tradeoff parameters *α*, *β*, *γ* and *τ*, the phase and intensity regularization terms can be written as:

14 $$\begin{array}{rcl}{\Gamma }_{{\rm{phase}}}(\varphi ) & = & \alpha \parallel \nabla \varphi {\parallel }_{1}+\beta (\parallel \nabla \varphi {\parallel }_{2}^{2}+\parallel {\nabla }^{2}\varphi {\parallel }_{2}^{2}),\\ {\Gamma }_{{\rm{intensity}}}(|\tilde{A}{|}^{2}) & = & \gamma (\parallel \nabla |\tilde{A}{|}^{2}{\parallel }_{1}+\parallel {\nabla }^{2}|\tilde{A}{|}^{2}{\parallel }_{1})+\tau (\parallel \nabla |\tilde{A}{|}^{2}{\parallel }_{2}^{2}+\parallel {\nabla }^{2}|\tilde{A}{|}^{2}{\parallel }_{2}^{2}).\end{array}$$

Γ_phase_(*ϕ*) and ${\Gamma }_{{\rm{intensity}}}(|\tilde{A}{|}^{2})$ contain only convolution operators or proxiable functions^67^, and hence Eq. (3) can be efficiently solved using primal-dual splitting methods such as a customized ADMM^54^ solver. Further mathematical and algorithmic details can be found in the Supplementary Materials. For normalized grayscale images valued between 0 and 255, typical tradeoff parameters are *α* = 0.1, *β* = 0.1, *γ* = 100, and *τ* = 5. A post-processing on final phase image is necessary in order to remove unwanted tilting artifacts. The ultimate goal of quantitative phase imaging is to find relative phase changes over time within a sample, so it is necessary to isolate the object relative to the background and prevent influence of the variations in the thickness of the coverslip or alignment of the sample relative to the microscope. To achieve this, a least-squares fitted affine plane to the recovered phase is subtracted from the phase estimation to remove undesired tilting artifacts. The whole algorithm was implemented in C++ and CUDA 10.0, and was run on a Ubuntu 18.04 workstation, equipped with Intel(R) Xeon(R) CPU E5-2680 @2.70 (2 × 16 cores), 62.9 GB memory, and a Nvidia GPU graphics card TITAN X (Pascal). Due to the iterative nature of the solver, we can trade off processing time vs. reconstruction quality. For an 1000 × 1000 pixel size raw image, with proper pre-caching of constant data (e.g. the reference image), the solver requires in ≈97 ms for 3 alternating iterations, and a full run of 10 alternating iterations takes ≈317 ms.

### Fabrication

The binary phase mask for the coded wavefront sensor was fabricated on a 0.5 mm thick 4″ Fused Silica wafer using photo-lithography techniques. The designed binary phase (either 0 or *π*) was converted to a binary mask pattern (either 0 or 1 and written on a photo-mask by a laser direct writer. Each pixel on the pattern is 12.9 μm. Accounting for diffraction, the frequency of resultant speckle field is within the sensor pixel sampling cutoff frequency. The fused silica wafer was deposited with a 200 nm thick Cr film after cleaning in piranha solution. Afterwards, the fused silica wafer with Cr was spin-coated with a uniform layer of photo-resist AZ1505 to form a 0.6 μm layer to be used in photolithography. The photo-mask and the wafer coated with photo-resist is then aligned for UV exposure. The exposed area on the photo-resist becomes soluble to the developer and can be removed. The design patterns were transferred to the photo-resist and the opening areas on the Cr film were then removed by Cr etchant, such that the mask patterns were transferred to Cr hard mask. Residual photo-resist was removed by ultrasonic rinse in acetone. Finally, the binary phase mask is obtained by etching the fused silica with mixed Argon and SF6 plasma. The fabricated mask is directly mounted on top of a monochromatic bare sensor (1501M-USB-TE, Thorlabs) by removing the original protection cover glass for which to be replaced with the mask. As such, the distance *z* between mask and the sensor plane is approximately 1.5 mm, but needs further calibration (see next subsection) to fully determine distance *z*, and hence the scale between actual pixel movements and real wavefront slopes.

### Calibration

According to Eq. (2), to correctly map from the numerically reconstructed surface to the original wavefront, an accurate calibration of the distance *z* is important and necessary. The exact distance is calibrated and characterized in another separate experiment as described in Fig. 8. This is accomplished by comparison between our numerical reconstruction wavefronts and the ground truth wavefronts. Figure 8(a) shows the optical setup, where a plasma broadband white light source (HPLS245, Thorlabs) is used for illumination. A pre-calibrated reflective phase-only spatial light modulator (SLM) (PLUTO-2-VIS-016, Holoeye) is configured to interpret grayscale images as 2*π* phase wrapping, for generating ground truth wavefronts. Some examples are shown in Fig. 8(b). A linear polarizer ensures the SLM operates in the pure phase modulation mode. The relay lenses (two *f* = 125 mm cemented achromatic doublets, AC254-125-A, Thorlabs) conjugate the SLM to the wavefront sensor plane at ×1 magnification ratio. By comparing the algorithm output wavefronts with the ground truth in Fig. 8(b), and with the known sensor pixel size 6.45 μm and SLM pixel size 8 μm, for each slope the calibrated distances are computed as in Fig. 8(c), where their mean is *z* = 1.43 mm.

### Samples

All human samples collected for these experiments are under a protocol approved by the Institutional Biosafety and Ethics Committee of King Abdullah University of Science and Technology with a waiver of consent.

#### Red blood cells preparation

Few of purified red blood cells from donors (NHGA blood bank Jeddah, Saudi Arabia) have been diluted in Phosphate buffer solution and placed between slides and cover slides.

#### HeLa cell culture

HeLa cells (ATCC CCL-2) were cultured in Minimum Essential Media (MEM, Gibco -Invitrogen, California U.S.A.) containing 10% Fetal Bovine Serum (FBS, Corning, New York, U.S.A.) and 1% HyClone Penicillin Streptomycin (Sigma-Aldrich, Missouri, U.S.A.). Cells were cultured at 37 °C in 5% CO_2_.

#### Cell culture and CLSM study

MCF-7 cells were seeded on coverslip at a density of 5 × 10^4^ cell/mL. Cells were cultured in DMEM medium containing 10% of FBS, 0.1% of penicillin-streptomycin, and supplemented with 1 × 10^−2^ mg/mL human recombinant insulin at 37 °C in a humidified 5% CO_2_ atmosphere. After cell attachment, cells were fixed with 4% of paraformaldehyde.

## Supplementary information

Supplementary Information

## Data Availability

The code and data used in this study are available on GitHub: https://github.com/PhaseIntensityMicroscope.
