# Supplementary material for: Quantitative Phase and Intensity Microscopy Using Snapshot White Light Wavefront Sensing
Source: Sci Rep. 2019 Sep 24;9:13795. doi: 10.1038/s41598-019-50264-3 (PMC6760235; doi:10.1038/s41598-019-50264-3)
Supplement: Supplementary file 1 — Supplementary Information [file 41598_2019_50264_MOESM1_ESM.pdf]

Supplementary Information:
Quantitative Phase and Intensity Microscopy Using Snapshot White Light Wavefront Sensing

Congli Wang[1], Qiang Fu[1], Xiong Dun[1], and Wolfgang Heidrich[1,*]

[1]Visual Computing Center, King Abdullah University of Science and Technology, Thuwal 23955-4700, Saudi Arabia
[*]wolfgang.heidrich@kaust.edu.sa

S.1 Theory

In this section we show how to extend previous speckle-pattern tracking phase retrieval techniques,[1–9] using two separate but consistent models, from physical optics and ray optics respectively, to derive a concise image formation model for our coded wavefront sensor.

Physical optics interpretation

Diffraction model

To minimize assumptions, our derivation is based on scalar diffraction theory in the form of the Rayleigh-Sommerfeld diffraction model, from which the well-known Fresnel diffraction formula can be derived. Consider a propagation problem where the scalar field $u_0(\mathbf{r})$ propagates through a short distance z and becomes another scalar field $u_z(\mathbf{r})$, where $\mathbf{r} = (x, y)$ represents the spatial coordinates. In the compact form of the Rayleigh-Sommerfeld formula,[10,11] the diffraction propagation can be written as:

$$u_z(\mathbf{r}) = \exp\left[\mathrm{j}kz \left(1 + \frac{\nabla^2}{k^2} \right)^{1/2} \right] u_0(\mathbf{r}) \quad \text{(spatial domain)} \qquad U_z(\boldsymbol{\rho}) = \exp\left[\mathrm{j}kz \left(1 - \lambda^2 |\boldsymbol{\rho}|^2 \right)^{1/2} \right] U_0(\boldsymbol{\rho}) \quad \text{(Fourier domain)}$$

where $\boldsymbol{\rho}$ is the Fourier dual of $\mathbf{r}$, and $U_0(\boldsymbol{\rho})$ and $U_z(\boldsymbol{\rho})$ are Fourier transforms of $u_0(\mathbf{r})$ and $u_z(\mathbf{r})$ respectively. Denote $\mathscr{F}$ as Fourier transform, then $U_0(\boldsymbol{\rho}) = \mathscr{F}\{u_0(\mathbf{r})\}$ and $U_z(\boldsymbol{\rho}) = \mathscr{F}\{u_z(\mathbf{r})\}$. λ is wavelength, $k = 2\pi/\lambda$ is wave number, and ∇^2 is the Laplacian operator. We follow conventional notation for wave optics, and let $|\cdot|$ denote the ℓ_2-norm of the vector. That said, mathematically $|\boldsymbol{\rho}| = (\boldsymbol{\rho}_x^2 + \boldsymbol{\rho}_y^2)^{1/2}$.

Diffraction from mask to sensor

Let the initial field $u_0(\mathbf{r})$ be the field at the mask plane. It is a multiplication of general (intensity or phase) modulation mask $p_0(\mathbf{r})$ and a general scalar field $f_0(\mathbf{r})$ that we would like to measure, i.e. $u_0(\mathbf{r}) = p_0(\mathbf{r}) f_0(\mathbf{r})$. At the sensor plane distance z away from the mask, the initial scalar field $u_0(\mathbf{r})$ gets diffracted as $u_z(\mathbf{r})$. In the following, small letters denote scalar fields in the spatial domain, whereas capital letters denote scalar fields in the Fourier domain, and z denotes the field at propagation distance. Using the Rayleigh-Sommerfeld formula and expanding the propagation in the Fourier domain, we have:[7]

$$\begin{aligned}
u_z(\mathbf{r}) &\triangleq \exp\left[\mathrm{j}kz \left(1 + \frac{\nabla^2}{k^2} \right)^{1/2} \right] u_0(\mathbf{r}) \\
&\overset{\mathscr{F}}{=} \int \exp(\mathrm{j}2\pi \mathbf{r}\cdot\boldsymbol{\rho}) \exp\left[\mathrm{j}kz \left(1 - \lambda^2 |\boldsymbol{\rho}|^2 \right)^{1/2} \right] \times \int P_0(\boldsymbol{\rho}') F_0(\boldsymbol{\rho} - \boldsymbol{\rho}')\, \mathrm{d}\boldsymbol{\rho}'\, \mathrm{d}\boldsymbol{\rho} \\
&\overset{\text{(S.2)}}{\approx} \exp(-\mathrm{j}kz) \int \exp(\mathrm{j}2\pi \mathbf{r}\cdot\boldsymbol{\rho}') \exp\left[\mathrm{j}kz \left(1 - \lambda^2 |\boldsymbol{\rho}'|^2 \right)^{1/2} \right] \times \\
&\qquad \int \exp\left[\mathrm{j}2\pi \left(\mathbf{r} - \lambda z \boldsymbol{\rho}' \right) \cdot \boldsymbol{\rho}'' \right] \exp\left[\mathrm{j}kz \left(1 - \lambda^2 |\boldsymbol{\rho}''|^2 \right)^{1/2} \right] F_0(\boldsymbol{\rho}'')\, \mathrm{d}\boldsymbol{\rho}'' P_0(\boldsymbol{\rho}')\, \mathrm{d}\boldsymbol{\rho}' \\
&= \exp(-\mathrm{j}kz) \int \exp(\mathrm{j}2\pi \mathbf{r}\cdot\boldsymbol{\rho}') P_z(\boldsymbol{\rho}') \times \left(\int \exp\left[\mathrm{j}2\pi \left(\mathbf{r} - \lambda z \boldsymbol{\rho}' \right) \cdot \boldsymbol{\rho}'' \right] F_z(\boldsymbol{\rho}'')\, \mathrm{d}\boldsymbol{\rho}'' \right) \mathrm{d}\boldsymbol{\rho}' \\
&\overset{\mathscr{F}^{-1}}{=} \exp(-\mathrm{j}kz) \int \exp(\mathrm{j}2\pi \mathbf{r}\cdot\boldsymbol{\rho}') P_z(\boldsymbol{\rho}') f_z(\mathbf{r} - \lambda z \boldsymbol{\rho}')\, \mathrm{d}\boldsymbol{\rho}',
\end{aligned} \tag{S.1}$$

where the third equality of Eq. (S.1) results from the introduction of variable $\boldsymbol{\rho}'' = \boldsymbol{\rho} - \boldsymbol{\rho}'$, and $\cdot$ denotes inner product. The approximation step comes from (for $\lambda = 500\,\text{nm}$ and pixel size $6.45\,\mu\text{m}$, at Nyquist frequency $\lambda^2|\boldsymbol{\rho}|^2 \sim 0.0015 \ll 1$):[10]

$$\left(1-\lambda^2|\boldsymbol{\rho}|^2\right)^{1/2} \approx \left(1-\lambda^2|\boldsymbol{\rho}'|^2\right)^{1/2} + \left(1-\lambda^2|\boldsymbol{\rho}''|^2\right)^{1/2} - \lambda^2\boldsymbol{\rho}'\cdot\boldsymbol{\rho}'' - 1. \tag{S.2}$$

To simplify notation, we neglect the constant phase term, and substitute notation $\boldsymbol{\rho}'$ with $\boldsymbol{\rho}$ in Eq. (S.1):

$$u_z(\mathbf{r}) = \int \exp(\mathrm{j}2\pi\mathbf{r}\cdot\boldsymbol{\rho}) P_z(\boldsymbol{\rho}) f_z(\mathbf{r}-\lambda z\boldsymbol{\rho})\,\mathrm{d}\boldsymbol{\rho}. \tag{S.3}$$

Note that Eq. (S.3) is a general formula and is applicable to any field that $u_0(\mathbf{r}) = p_0(\mathbf{r}) f_0(\mathbf{r})$. Wang et al.[7] got their formula by further approximating Eq. (S.3) by:

1. Ignoring the diffraction of field $f_0(\mathbf{r}-\lambda z\boldsymbol{\rho})$, i.e. $f_z(\mathbf{r}-\lambda z\boldsymbol{\rho}) \approx f_0(\mathbf{r}-\lambda z\boldsymbol{\rho})$.
2. Considering uniform amplitude case where $f_0(\mathbf{r}) = \exp[\mathrm{j}\phi(\mathbf{r})]$ and $\phi(\mathbf{r})$ is the desired wavefront to be recovered.
3. Linearizing phase $\phi(\mathbf{r}-\lambda z\boldsymbol{\rho})$, and preserving only the first-order term, i.e. $\phi(\mathbf{r}-\lambda z\boldsymbol{\rho}) \approx \phi(\mathbf{r}) - \lambda z\boldsymbol{\rho}\cdot\nabla\phi(\mathbf{r})$.

In this Supplementary, we preserve the 3rd approximation, but re-formulate the 1st approximation by considering the small diffraction of the field $f_0(\mathbf{r}-\lambda z\boldsymbol{\rho})$, and relax the 2nd approximation to be a general field that $f_0(\mathbf{r}) = A(\mathbf{r})\exp[\mathrm{j}\phi(\mathbf{r})]$ where $A(\mathbf{r})$ is the sample amplitude that we would also like to recover. We now discuss our reformulations on works of Wang et al.[7]

Sample diffraction

Consider $f_z(\mathbf{r})$, the diffraction field of $f_0(\mathbf{r})$ at distance z. Since scalar field $f_0(\mathbf{r}) = A(\mathbf{r})\exp[\mathrm{j}\phi(\mathbf{r})]$ is the product of the amplitude and the phase exponential, by Eq. (S.3), we can decompose $f_z(\mathbf{r})$ in terms of its amplitude and phase contributions:

$$f_z(\mathbf{r}) = \int \exp(\mathrm{j}2\pi\mathbf{r}\cdot\boldsymbol{\rho}) \mathscr{A}_z(\boldsymbol{\rho}) g_z(\mathbf{r}-\lambda z\boldsymbol{\rho})\,\mathrm{d}\boldsymbol{\rho}, \tag{S.4}$$

where $\mathscr{A}_z(\boldsymbol{\rho})$ is the Fourier transform of $A_z(\mathbf{r})$, $A_z(\mathbf{r})$ and $g_z(\mathbf{r})$ are the diffraction fields of $A(\mathbf{r})$ and $g(\mathbf{r}) = \exp[\mathrm{j}\phi(\mathbf{r})]$ respectively. We now discuss how to compute $A_z(\mathbf{r})$ (the amplitude diffraction field) and $g_z(\mathbf{r})$ (the phase diffraction field), respectively.

Amplitude diffraction field. Under Fresnel diffraction assumption, denote $*$ as convolution, the diffraction field $A_z(\mathbf{r})$ is computed as (neglecting the obliquity factor):[11]

$$\begin{aligned} A_z(\mathbf{r}) &\triangleq A(\mathbf{r}) * \frac{\exp(\mathrm{j}kz)}{\mathrm{j}\lambda z}\exp\left(\mathrm{j}\frac{\pi|\mathbf{r}|^2}{\lambda z}\right) \\ &\triangleq \frac{\exp(\mathrm{j}kz)}{\mathrm{j}\lambda z}\int A(\mathbf{r}-\mathbf{r}')\exp\left(\mathrm{j}\frac{\pi|\mathbf{r}'|^2}{\lambda z}\right)\mathrm{d}\mathbf{r}', \end{aligned} \tag{S.5}$$

which can be further simplified by preserving only the 0th order of $A(\mathbf{r}-\mathbf{r}')$, i.e. $A(\mathbf{r}-\mathbf{r}') \approx A(\mathbf{r})$ (see also Fig. S.1 for explanation), and neglecting the constant phase terms:

$$\begin{aligned} A_z(\mathbf{r}) &\approx \int A(\mathbf{r})\exp\left(\mathrm{j}\frac{\pi|\mathbf{r}'|^2}{\lambda z}\right)\mathrm{d}\mathbf{r}' \\ &\overset{\text{(S.56)}}{=} \frac{A(\mathbf{r})}{\sqrt{\pi}}\exp\left(\mathrm{j}\frac{\pi}{4}\right), \end{aligned} \tag{S.6}$$

where we utilize the Fourier transform pair formula (Eq. (S.56)) that can be found in the Appendix. Consequently we have:

$$A_z(\mathbf{r}) = A(\mathbf{r}) \quad \text{(spatial domain)} \qquad \mathscr{A}_z(\boldsymbol{\rho}) = \mathscr{A}(\boldsymbol{\rho}) \quad \text{(Fourier domain)}, \tag{S.7}$$

where we neglect all the constant factors, including $1/\sqrt{\pi}$, and $\mathscr{A}(\boldsymbol{\rho})$ denotes the Fourier transform of $A(\mathbf{r})$. Equation (S.7) is intuitive: for a zero phase amplitude field, it should preserve its amplitude and remain the null phase status in a sufficiently short propagation distance z.

Phase diffraction field. Similarly, by Fresnel diffraction formula, the diffraction field of $g(\mathbf{r})$ is computed as:

$$
\begin{aligned}
g_z(\mathbf{r}) &\triangleq \exp[\mathrm{j}\phi(\mathbf{r})] * \frac{\exp(\mathrm{j}kz)}{\mathrm{j}\lambda z} \exp\left(\mathrm{j}\frac{\pi|\mathbf{r}|^2}{\lambda z}\right) \\
&\triangleq \frac{\exp(\mathrm{j}kz)}{\mathrm{j}\lambda z} \int \exp[\mathrm{j}\phi(\mathbf{r}-\mathbf{r}')] \exp\left(\mathrm{j}\frac{\pi|\mathbf{r}'|^2}{\lambda z}\right) \mathrm{d}\mathbf{r}'. \qquad \text{(S.8)}
\end{aligned}
$$

A Taylor expansion of $\phi(\mathbf{r}-\mathbf{r}')$ around $\mathbf{r}$ up to second order suggests:

$$
\phi(\mathbf{r}-\mathbf{r}') \approx \phi(\mathbf{r}) - \nabla\phi(\mathbf{r})\cdot\mathbf{r}' + \frac{1}{2}\nabla^2\phi(\mathbf{r})\cdot|\mathbf{r}'|^2. \qquad \text{(S.9)}
$$

With the approximation in Eq. (S.9), Eq. (S.8) is simplified as:

$$
\begin{aligned}
g_z(\mathbf{r}) &\overset{\text{(S.8)}}{=} \int \exp[\mathrm{j}\phi(\mathbf{r}-\mathbf{r}')] \exp\left(\mathrm{j}\frac{\pi|\mathbf{r}'|^2}{\lambda z}\right) \mathrm{d}\mathbf{r}' \\
&\overset{\text{(S.9)}}{\approx} \exp[\mathrm{j}\phi(\mathbf{r})] \int \exp[-\mathrm{j}\nabla\phi(\mathbf{r})\cdot\mathbf{r}'] \exp\left[\mathrm{j}|\mathbf{r}'|^2\left(1+\frac{\lambda z}{2\pi}\nabla^2\phi(\mathbf{r})\right)\right] \mathrm{d}\mathbf{r}' \\
&\overset{\text{(S.56)}}{=} \frac{\exp[\mathrm{j}\phi(\mathbf{r})]}{\sqrt{\pi\left(1+\frac{\lambda z}{2\pi}\nabla^2\phi(\mathbf{r})\right)}} \underbrace{\exp\left(\mathrm{j}\frac{\pi - \frac{1}{\left(1+\frac{\lambda z}{2\pi}\nabla^2\phi(\mathbf{r})\right)}|\nabla\phi(\mathbf{r})|^2}{4}\right)}_{\exp[\mathrm{j}C(\phi)]}, \qquad \text{(S.10)}
\end{aligned}
$$

where $C(\phi)$ is a phase function of $\phi(\mathbf{r})$. Consequently we have:

$$
g_z(\mathbf{r}) = \frac{1}{\sqrt{1+\frac{\lambda z}{2\pi}\nabla^2\phi(\mathbf{r})}} \exp[\mathrm{j}\phi(\mathbf{r})] \exp[\mathrm{j}C(\phi)], \qquad \text{(S.11)}
$$

where we neglect the constant factor $1/\sqrt{\pi}$. Equation (S.11) indicates that, though starting with a uniform amplitude, there is a small amplitude change for the diffraction field of the phase exponential term $g(\mathbf{r})$ due to local wavefront curvature $\nabla^2\phi(\mathbf{r})$.

Total diffraction field. Given results from Eq. (S.7) and Eq. (S.11), we can calculate $f_z(\mathbf{r})$ in Eq. (S.4) as:

$$
\begin{aligned}
f_z(\mathbf{r}) &\overset{\text{(S.4)}}{=} \int \exp(\mathrm{j}2\pi\mathbf{r}\cdot\boldsymbol{\rho}) \mathscr{A}_z(\boldsymbol{\rho}) g_z(\mathbf{r}-\lambda z\boldsymbol{\rho})\,\mathrm{d}\boldsymbol{\rho} \\
&\underset{\text{(S.11)}}{\overset{\text{(S.7)}}{=}} \int \exp(\mathrm{j}2\pi\mathbf{r}\cdot\boldsymbol{\rho}) \frac{\mathscr{A}(\boldsymbol{\rho})}{\sqrt{1+\frac{\lambda z}{2\pi}\nabla^2\phi(\mathbf{r}-\lambda z\boldsymbol{\rho})}} \exp[\mathrm{j}\phi(\mathbf{r}-\lambda z\boldsymbol{\rho})] \exp[\mathrm{j}C(\phi(\mathbf{r}-\lambda z\boldsymbol{\rho}))]\,\mathrm{d}\boldsymbol{\rho} \\
&\overset{\text{(S.13)}}{\approx} \int \exp(\mathrm{j}2\pi\mathbf{r}\cdot\boldsymbol{\rho}) \frac{\mathscr{A}(\boldsymbol{\rho})}{\sqrt{1+\frac{\lambda z}{2\pi}\nabla^2\phi(\mathbf{r})}} \exp[\mathrm{j}\phi(\mathbf{r}-\lambda z\boldsymbol{\rho})] \exp[\mathrm{j}C(\phi(\mathbf{r})]\,\mathrm{d}\boldsymbol{\rho}, \qquad \text{(S.12)}
\end{aligned}
$$

where we preserve only the 0th order of the terms in $\nabla^2\phi(\mathbf{r})$ and $C(\phi(\mathbf{r}))$, i.e. assuming zero frequency shifts for these two minor terms due to $\boldsymbol{\rho} = \mathbf{0}$:

$$
\nabla^2\phi(\mathbf{r}\pm\lambda z\boldsymbol{\rho}) \approx \nabla^2\phi(\mathbf{r}) \qquad \text{and} \qquad C(\phi(\mathbf{r}\pm\lambda z\boldsymbol{\rho})) \approx C(\phi(\mathbf{r})). \qquad \text{(S.13)}
$$

Recall the 3rd approximation mentioned previously in Wang et al.[7] that:

$$
\phi(\mathbf{r}-\lambda z\boldsymbol{\rho}) \approx \phi(\mathbf{r}) - \lambda z\boldsymbol{\rho}\cdot\nabla\phi(\mathbf{r}). \qquad \text{(S.14)}
$$

Therefore, Eq. (S.12) can be further simplified to:

$$
\begin{aligned}
f_z(\mathbf{r}) &\overset{\text{(S.14)}}{\approx} \int \exp\left[\mathrm{j}2\pi\left(\mathbf{r}-\frac{\lambda z}{2\pi}\nabla\phi\right)\cdot\boldsymbol{\rho}\right] \frac{\mathscr{A}(\boldsymbol{\rho})}{\sqrt{1+\frac{\lambda z}{2\pi}\nabla^2\phi(\mathbf{r})}} \exp[\mathrm{j}\phi(\mathbf{r})] \exp[\mathrm{j}C(\phi(\mathbf{r}))]\,\mathrm{d}\boldsymbol{\rho} \\
&= \frac{\exp[\mathrm{j}\phi(\mathbf{r})]\exp[\mathrm{j}C(\phi(\mathbf{r}))]}{\sqrt{1+\frac{\lambda z}{2\pi}\nabla^2\phi(\mathbf{r})}} \int \exp\left[\mathrm{j}2\pi\left(\mathbf{r}-\frac{\lambda z}{2\pi}\nabla\phi\right)\cdot\boldsymbol{\rho}\right] \mathscr{A}(\boldsymbol{\rho})\,\mathrm{d}\boldsymbol{\rho} \\
&\overset{\mathscr{F}^{-1}}{=} \frac{\exp[\mathrm{j}\phi(\mathbf{r})]\exp[\mathrm{j}C(\phi(\mathbf{r}))]}{\sqrt{1+\frac{\lambda z}{2\pi}\nabla^2\phi(\mathbf{r})}} A\left(\mathbf{r}-\frac{\lambda z}{2\pi}\nabla\phi\right). \qquad \text{(S.15)}
\end{aligned}
$$

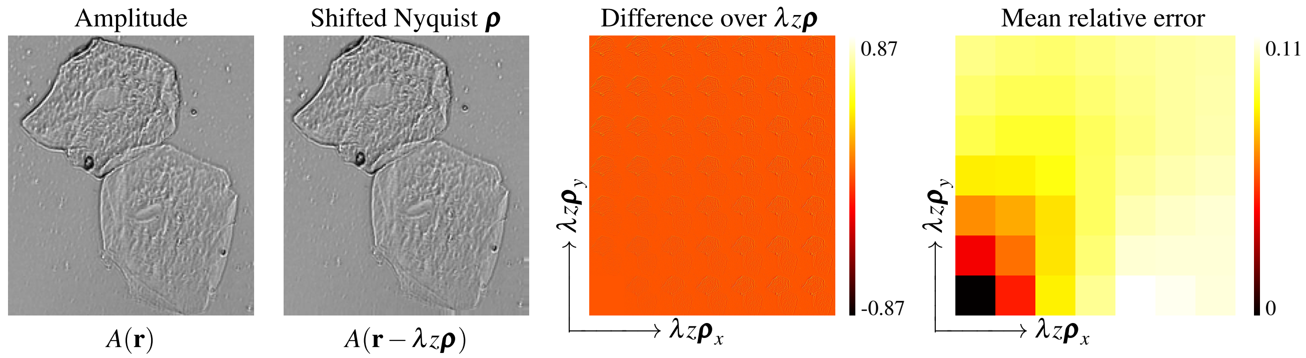

Figure S.1. Relatively flat smooth specimen amplitude ensures a small amplitude gradient that can be ignored in Eq. (S.16). From left to right: Original square-rooted bright field image, i.e. the amplitude $A(\mathbf{r})$, normalized between 0 and 1; One of the many numerically shifted images are shown, which is at Nyquist frequency $\boldsymbol{\rho}$ (most shifts); Difference map over ρ; Mean relative error for each shift. In most areas, the difference between $A(\mathbf{r})$ and $A(\mathbf{r}-\lambda z\boldsymbol{\rho})$ is small, therefore the approximation that $A(\mathbf{r}) \approx A(\mathbf{r}-\lambda z\boldsymbol{\rho})$ is valid within Nyquist frequency $\boldsymbol{\rho}$.

Based on Eq. (S.15), we can compute the total diffraction field $f_z(\mathbf{r}-\lambda z\boldsymbol{\rho})$ as:

$$
\begin{aligned}
f_z(\mathbf{r}-\lambda z\boldsymbol{\rho}) &\overset{(\text{S.15})}{=} \frac{\exp[\mathrm{j}C(\phi(\mathbf{r}-\lambda z\boldsymbol{\rho}))]}{\sqrt{1+\frac{\lambda z}{2\pi}\nabla^2\phi(\mathbf{r}-\lambda z\boldsymbol{\rho})}} \exp[\mathrm{j}\phi(\mathbf{r}-\lambda z\boldsymbol{\rho})] A\left(\mathbf{r}-\lambda z\boldsymbol{\rho}-\frac{\lambda z}{2\pi}\nabla\phi\right) \\
&\overset{(\text{S.13})}{\approx} \frac{\exp[\mathrm{j}C(\phi(\mathbf{r}))]}{\sqrt{1+\frac{\lambda z}{2\pi}\nabla^2\phi(\mathbf{r})}} \exp[\mathrm{j}\phi(\mathbf{r}-\lambda z\boldsymbol{\rho})] A\left(\mathbf{r}-\lambda z\boldsymbol{\rho}-\frac{\lambda z}{2\pi}\nabla\phi\right),
\end{aligned} \tag{S.16}
$$

where again we preserve only the 0th order of $\nabla^2\phi(\mathbf{r})$ and $C(\phi(\mathbf{r}))$.

Amplitude image approximation. We now argue that $A(\mathbf{r}-\lambda z\boldsymbol{\rho}-\frac{\lambda z}{2\pi}\nabla\phi) \approx A(\mathbf{r}-\frac{\lambda z}{2\pi}\nabla\phi)$ in Eq. (S.16). In many phase imaging applications the specimens have small amplitude variations, i.e. the amplitude gradient is small. Unfortunately the spatial shift $\lambda z\boldsymbol{\rho}$ is usually too large and is beyond the convergence range of Taylor expansion. Constant pixel shift $\lambda z/(2\pi)\nabla\phi$ does not change the relative positions, so for simplicity we can safely analyze $A(\mathbf{r})$ and $A(\mathbf{r}-\lambda z\boldsymbol{\rho})$ instead. To perform a quantitative evaluation, we visualize $A(\mathbf{r})$ and $A(\mathbf{r}-\lambda z\boldsymbol{\rho})$ in Fig. S.1, and compute the differences between them with different shift amounts $\lambda z\boldsymbol{\rho}$. Given $\lambda = 500\,\text{nm}$, $z = 1\,\text{mm}$, pixel size $6.45\,\mu\text{m}$, the maximum shift pixel number is ~ 6 pixels at Nyquist frequency $|\boldsymbol{\rho}| = 1/12.9\,\mu\text{m}^{-1}$. Relative errors between the shifts are small.

Therefore in Eq. (S.16), we can safely approximate $A(\mathbf{r}-\lambda z\boldsymbol{\rho}-\frac{\lambda z}{2\pi}\nabla\phi)$ as $A(\mathbf{r}-\frac{\lambda z}{2\pi}\nabla\phi)$. Further, by applying the linearization (i.e. Eq. (S.14)) again, we obtain:

$$
\begin{aligned}
f_z(\mathbf{r}-\lambda z\boldsymbol{\rho}) &\approx \frac{\exp[\mathrm{j}C(\phi(\mathbf{r}))]}{\sqrt{1+\frac{\lambda z}{2\pi}\nabla^2\phi(\mathbf{r})}} \exp[\mathrm{j}\phi(\mathbf{r}-\lambda z\boldsymbol{\rho})] A\left(\mathbf{r}-\frac{\lambda z}{2\pi}\nabla\phi\right) \\
&\overset{(\text{S.14})}{\approx} \frac{\exp[\mathrm{j}C(\phi(\mathbf{r}))]}{\sqrt{1+\frac{\lambda z}{2\pi}\nabla^2\phi(\mathbf{r})}} \exp[\mathrm{j}\phi(\mathbf{r})] \exp[-\mathrm{j}\lambda z\boldsymbol{\rho}\cdot\nabla\phi(\mathbf{r})] A\left(\mathbf{r}-\frac{\lambda z}{2\pi}\nabla\phi\right).
\end{aligned} \tag{S.17}
$$

Final result

The derived Eq. (S.17) leads to the following final formula for Eq. (S.3):

$$
\begin{aligned}
u_z(\mathbf{r}) &\overset{(\text{S.3})}{=} \int \exp(\mathrm{j}2\pi\mathbf{r}\cdot\boldsymbol{\rho}) P_z(\boldsymbol{\rho}) f_z(\mathbf{r}-\lambda z\boldsymbol{\rho})\,\mathrm{d}\boldsymbol{\rho} \\
&\overset{(\text{S.17})}{=} \frac{A\left(\mathbf{r}-\frac{\lambda z}{2\pi}\nabla\phi\right)}{\sqrt{1+\frac{\lambda z}{2\pi}\nabla^2\phi}} \int \exp\left[\mathrm{j}2\pi\left(\mathbf{r}-\frac{\lambda z}{2\pi}\nabla\phi\right)\cdot\boldsymbol{\rho}\right] P_z(\boldsymbol{\rho})\,\mathrm{d}\boldsymbol{\rho} \\
&\overset{\mathscr{F}^{-1}}{=} \frac{A\left(\mathbf{r}-\frac{\lambda z}{2\pi}\nabla\phi\right)}{\sqrt{1+\frac{\lambda z}{2\pi}\nabla^2\phi}} p_z\left(\mathbf{r}-\frac{\lambda z}{2\pi}\nabla\phi\right),
\end{aligned} \tag{S.18}
$$

where we neglect all the constant phase terms in Eq. (S.17). The intensity image received on the sensor will then be:

$$\begin{aligned}
I(\mathbf{r}) &\triangleq |u_z(\mathbf{r})|^2 \\
&\overset{(S.18)}{=} \frac{\left|A\left(\mathbf{r}-\frac{\lambda z}{2\pi}\nabla\phi\right)\right|^2}{1+\frac{\lambda z}{2\pi}\nabla^2\phi}\left|p_z\left(\mathbf{r}-\frac{\lambda z}{2\pi}\nabla\phi\right)\right|^2 \\
&\overset{|\nabla^2\phi|\ll\frac{2\pi}{\lambda z}}{\approx} \left|A\left(\mathbf{r}-\frac{\lambda z}{2\pi}\nabla\phi\right)\right|^2\left(1-\frac{\lambda z}{2\pi}\nabla^2\phi\right)\left|p_z\left(\mathbf{r}-\frac{\lambda z}{2\pi}\nabla\phi\right)\right|^2 \\
&= \left|A\left(\mathbf{r}-\frac{\lambda z}{2\pi}\nabla\phi\right)\right|^2\left(1-\frac{\lambda z}{2\pi}\nabla^2\phi\right)I_0\left(\mathbf{r}-\frac{\lambda z}{2\pi}\nabla\phi\right),
\end{aligned} \tag{S.19}$$

where we approximate the denominator with first order Taylor expansion in the reason that $\lambda z/(2\pi)|\nabla^2\phi| \ll 1$, and a reference image $I_0(\mathbf{r}) = |p_z(\mathbf{r})|^2$ is taken under collimated illumination that $\phi_0(\mathbf{r}) =$ constant. Equation (S.19) can be re-arranged as:

$$\boxed{I\left(\mathbf{r}+\frac{\lambda z}{2\pi}\nabla\phi\right) = |A(\mathbf{r})|^2\left(1-\frac{\lambda z}{2\pi}\nabla^2\phi\right)I_0(\mathbf{r})} \tag{S.20}$$

Notice Eq. (S.20) can be reformulated in terms of optical path differences (OPD), i.e. with no wavelength λ. That said, temporally coherent light is not necessary and our sensor can work under broadband illumination, which is also verified in the experiments. Further, to convert from OPD to wavefront/phase, a nominal wavelength (e.g. $\lambda = 550\,\text{nm}$) could be used, as been normally seen in other slopes-tracking white light wavefront sensing applications such as Shack-Hartmann. Equation (S.20) is the main result under a physical optics model, and is the principle behind our wavefront sensor.

When under un-collimated illumination, i.e. $\phi_0 \neq 0$ and $\nabla^2\phi_0 \neq 0$, Eq. (S.20) is modified as:

$$\begin{aligned}
I\left(\mathbf{r}+\frac{\lambda z}{2\pi}\nabla\phi\right) &\approx |A(\mathbf{r})|^2\left(1-\frac{\frac{\lambda z}{2\pi}\nabla^2\phi}{1-\frac{\lambda z}{2\pi}\nabla^2\phi_0}\right)I_0(\mathbf{r}) \\
&\approx |A(\mathbf{r})|^2\left(1-\frac{\lambda z}{2\pi}\nabla^2\phi\right)I_0(\mathbf{r}),
\end{aligned} \tag{S.21}$$

where the first approximation comes from the amplitude image approximation (Fig. S.1), and the second approximation ignores the higher order terms, given that $|\frac{\lambda z}{2\pi}\nabla^2\phi_0| \ll 1$. For simple validation, for instance, at wavelength $\lambda = 500\,\text{nm}$, an 100 mm radius paraxial spherical wavefront satisfies $|\frac{\lambda z}{2\pi}\nabla^2\phi_0| = 0.015 \ll 1$ for sensor-mask distance $z = 1.5\,\text{mm}$, indicating a practical engineering tolerance for un-collimated illumination beams. As a result, we can safely conclude that in Eq. (S.20), for practical setups, the reference illumination does not affect subsequent measurements as long as both the reference image and measurement image are taken under the same illumination configuration.

Compared to the original formula in Wang et al.[7], the obtained more general Eq. (S.20) contains an additional multiplication of the sample intensity $|A(\mathbf{r})|^2$, and a higher order intensity change due to the wavefront curvature $\nabla^2\phi$. Equation (S.20) indicates that, when neglecting the intensity change by the wavefront curvature, the specimen intensity $|A(\mathbf{r})|^2$ and phase $\phi(\mathbf{r})$ are separately encoded in the intensity term and in the coordinate term. Simultaneous intensity and phase reconstruction is possible via a numerical optimization algorithm that will be discussed in Section S.2.

Ray optics interpretation

Damberg and Heidrich[12] proposed a warping based phase optimization image formation model for computational lens design, in which the core idea is that the energy conservation law is valid in each local differentiable area, and consequently a ray optics based formula can be derived for caustic image formation from a freeform lens. Here, we show how their idea can be elegantly revived as a ray optics perspective to our work. Their (approximated) image formation model, modified by taking the modulation mask diffraction into consideration, at wavelength λ, in our notation, at the calibration phase, is written as (refer Eq. (S.61) in Appendix for a short re-derivation):

$$I_0\left(\mathbf{r}+\frac{\lambda z}{2\pi}\nabla\phi_0\right) = \left(1-\frac{\lambda z}{2\pi}\nabla^2\phi_0\right)J(\mathbf{r}), \tag{S.22}$$

where $J(\mathbf{r})$ is the diffraction pattern (of the modulation mask) at the sensor plane, containing contribution from background illumination. That said, $J(\mathbf{r})$ has taken the consideration of mask diffraction. And $\phi_0(\mathbf{r})$ is the initial wavefront from the

background illumination. At the measurement phase, when there is unknown intensity $|A(\mathbf{r})|^2$ and phase $\phi(\mathbf{r})$, the measurement image becomes:

$$I\left(\mathbf{r}+\frac{\lambda z}{2\pi}\nabla(\phi_0+\phi)\right)=\left[1-\frac{\lambda z}{2\pi}\nabla^2(\phi_0+\phi)\right]J(\mathbf{r})|A(\mathbf{r})|^2. \tag{S.23}$$

With the introduction of auxiliary variable $\mathbf{r}'=\mathbf{r}+\lambda z/(2\pi)\nabla\phi_0$ and Eq. (S.22), Eq. (S.23) can be re-phased as:

$$\begin{aligned} I\left(\mathbf{r}'+\frac{\lambda z}{2\pi}\nabla\phi\right) &\overset{(S.23)}{=} |A(\mathbf{r})|^2\left(I_0(\mathbf{r}')-\frac{\lambda z}{2\pi}\nabla^2\phi J(\mathbf{r})\right) \\ &\overset{(S.22)}{=} |A(\mathbf{r})|^2\left(1-\frac{c}{1-c}\right)I_0(\mathbf{r}') \\ &\overset{c\ll 1}{\approx} |A(\mathbf{r})|^2\left(1-c-c^2\right)I_0(\mathbf{r}') \\ &\approx |A(\mathbf{r})|^2(1-c)\,I_0(\mathbf{r}'), \end{aligned} \tag{S.24}$$

where $c=\frac{\lambda z}{2\pi}|\nabla^2\phi|\ll 1$ and is valid for the approximations. When under collimated illumination, $\phi_0=$ constant, and we have $\mathbf{r}'=\mathbf{r}$ and $I_0(\mathbf{r})=J(\mathbf{r})$. Therefore:

$$I\left(\mathbf{r}+\frac{\lambda z}{2\pi}\nabla\phi\right)=|A(\mathbf{r})|^2\left(1-\frac{\lambda z}{2\pi}\nabla^2\phi\right)I_0(\mathbf{r}). \tag{S.25}$$

Equation (S.25) is the main result under ray optics model, and is a natural consequence from the Damberg & Heidrich model. Notice the equivalence between Eq. (S.25) and Eq. (S.20).

Spatial wavefront resolution analysis

Given the main result Eq. (S.20), we would like to know the maximum spatial wavefront resolution that it can resolve. As mentioned before, our sensor is a slopes-tracking sensor. When speckles are overlapped with each other, it is ambiguous for the solver to perform wavefront retrieval. To avoid this scenario, the wrapped coordinate in the measurement image I should be monotonically increasing or decreasing. Mathematically:

$$\frac{\partial}{\partial\mathbf{r}}\left(\mathbf{r}\pm\frac{\lambda z}{2\pi}\nabla\phi\right)\geq\mathbf{0} \quad\Rightarrow\quad \mp\frac{\lambda z}{2\pi}\nabla^2\phi\leq 1 \quad\overset{\phi=\lambda/(2\pi)\cdot\text{OPD}}{\Rightarrow}\quad \left|\nabla^2\text{OPD}\right|\leq\frac{1}{z} \quad\overset{|\nabla^2\phi|\ll 2\pi/(\lambda z)}{\Rightarrow}\quad \left|\nabla^2\text{OPD}\right|\ll\frac{1}{z}. \tag{S.26}$$

This conclusion is consistent with the small wavefront curvature assumption that $\lambda z/(2\pi)\cdot|\nabla^2\phi|\ll 1$ when deriving Eq. (S.20) from Eq. (S.19). Similar analysis has been done in terms of Fourier harmonic functions in Berto et al.[8], yet without specifying the small wavefront curvature assumption, and hence their bound ($\left|\nabla^2\text{OPD}\right|\leq 1/z$) is not as tight as ours ($\left|\nabla^2\text{OPD}\right|\ll 1/z$). Please refer to the main text for our measurement of the actual bound, and the resultant wavefront resolution reduction.

Connection to the Transport-of-Intensity Equation (TIE)

The well-known Transport-of-Intensity Equation (TIE), a.k.a. the irradiance transport equation, has been one of the norms for determined phase retrieval since its discovery back in early 1980s.[10,13–21] Here, we show the famous TIE can be derived from the principle revealed here, i.e. from Eq. (S.20). If doing Taylor expansion for $I(\mathbf{r}+\frac{\lambda z}{2\pi}\nabla\phi)$ around $\mathbf{r}$, by preserving only the first order, and re-arranging the terms, we have:

$$\nabla I(\mathbf{r})\cdot\nabla\phi+|A(\mathbf{r})|^2I_0(\mathbf{r})\nabla^2\phi=\frac{k}{z}\Big(|A(\mathbf{r})|^2I_0(\mathbf{r})-I(\mathbf{r})\Big). \tag{S.27}$$

In traditional TIE setups, there is no masks, and hence $I_0(\mathbf{r})=1$. To see it more clearly, let the image captured at the original mask plane be $I_1(\mathbf{r})=|A(\mathbf{r})|^2$, and the second image captured at the original sensor plane be $I_2(\mathbf{r})=I(\mathbf{r})$, then Eq. (S.27) can be reformulated as:

$$\nabla I_2\cdot\nabla\phi+I_1\nabla^2\phi=\frac{k}{z}(I_1-I_2)\approx -k\frac{\partial\bar{I}}{\partial z}. \tag{S.28}$$

Let $I_1(\mathbf{r})\approx I_2(\mathbf{r})\approx\bar{I}(\mathbf{r})$ we arrive at the standard form of TIE, when $z\to 0$, justifying the finite difference approximation. The derivation from Eq. (S.20) to TIE (i.e. Eq. (S.28)) shows that TIE is a special case of our model, however, our model is more powerful than TIE in a number of ways:

- A concise theoretical model for two distanced planes, which can be separated far away (e.g. $z = 1\,\text{mm}$), whereas TIE is only valid at one particular transversal plane, and suffers from finite approximation for the z-axis derivative. As such, the distance control has to be careful and precise, for example at μm scale. This allows our sensor to outperform in terms of sensitivity and setup easiness.
- Our model contains an image warping operation, which preserves nonlinearity and can be linearized (if desired) to match the linear formulations in TIE.
- Our model extends the degrees of freedom for classical TIE systems to allow for a customized-made modulation mask (reflected as a customizable reference image $I_0(\mathbf{r})$, for which in TIE is usually uniform), thus making it possible for single-shot measurements.
- Based on our model, a much noise-robust (that ignores the wavefront curvature at first) while efficient algorithm can be derived with modern optimization frameworks to estimate the unknowns. Please see Section S.2 for more numerical and algorithmic details.
- Our model allows for broadband illumination because it is formulated in terms of optical path differences, while TIE in principle requires coherent light.

Connection to previous works and their models

In the absence of from light ray attenuation, previous works in speckle-pattern tracking can be grouped into different forms of Eq. (S.20), as been summarized in Table S.1. We now describe each model followed by a short discussion.

Table S.1. Theoretical models used in previous speckle-pattern tracking research.

	Model	Articles
Flow-tracking	$I\left(\mathbf{r}+\frac{\lambda z}{2\pi}\nabla\phi\right) = I_0(\mathbf{r})$	[1,2,7,8,22–26]
Amplitude-contained flow-tracking	$I\left(\mathbf{r}+\frac{\lambda z}{2\pi}\nabla\phi\right) = \lvert A(\mathbf{r})\rvert^2 I_0(\mathbf{r})$ or $I(\mathbf{r}) = \lvert A(\mathbf{r})\rvert^2 I_0\left(\mathbf{r}-\frac{\lambda z}{2\pi}\nabla\phi\right)$	[4,27]
Eq. (S.20)	$I\left(\mathbf{r}+\frac{\lambda z}{2\pi}\nabla\phi\right) = \lvert A(\mathbf{r})\rvert^2 \left(1-\frac{\lambda z}{2\pi}\nabla^2\phi\right) I_0(\mathbf{r})$	[12]

Flow-tracking model

If we drop out the amplitude term and the wavefront curvature term, from Eq. (S.20) one has:

$$I\left(\mathbf{r}+\frac{\lambda z}{2\pi}\nabla\phi\right) = I_0(\mathbf{r}), \tag{S.29}$$

which we recognize as the famous optical flow formulation in computer vision,[28,29] or the previous tracking-based speckle-pattern phase reconstruction techniques.[1,2,7,8,22,23] Now we show lateral shearing interferometry wavefront sensing[24–26] as one special variant to this linear displacement model as following. For lateral shearing interferometers the mask is usually a specially designed grating, for simplicity here we only discuss the orthogonal sinusoidal grating in Bon et al.[25], other gratings can be handled in a similar way. Say with a spatial frequency $\omega/2$ the mask transmission function is $p(\mathbf{r}) = \cos(\omega x/2)\cos(\omega y/2)$. The intensity image under collimated illumination would be:

$$I_0(\mathbf{r}) = [1-\cos(\omega x)]\cdot[1-\cos(\omega y)] = 1-\cos(\omega x)-\cos(\omega y)+\cos(\omega x)\cos(\omega y). \tag{S.30}$$

Under a distorted wavefront $\phi(\mathbf{r})$ and negligible background illumination change, the received image gets local frequency shift due to local wavefront slopes. Shifting Eq. (S.29) we have:

$$\begin{aligned} I(\mathbf{r}) &\overset{(S.29)}{=} I_0\left(\mathbf{r}-\frac{\lambda z}{2\pi}\nabla\phi\right) \\ &\overset{(S.30)}{=} 1-\underbrace{\left(\cos\left[\omega\left(x-\frac{\lambda z}{2\pi}\nabla_x\phi\right)\right]+\cos\left[\omega\left(y-\frac{\lambda z}{2\pi}\nabla_y\phi\right)\right]\right)}_{I_{\text{slopes}}(\mathbf{r}),\ \text{the wavefront slopes encoded terms}}+\cos\left[\omega\left(x-\frac{\lambda z}{2\pi}\nabla_x\phi\right)\right]\cos\left[\omega\left(y-\frac{\lambda z}{2\pi}\nabla_y\phi\right)\right]. \end{aligned} \tag{S.31}$$

By interferogram analysis[30,31], the wavefront slopes $\nabla\phi$ are encoded in $I_{\text{slopes}}(\mathbf{r})$, and notice the assumption that above terms are separable (non-overlapping) in Fourier domain. If only $I_{\text{slopes}}(\mathbf{r})$ is considered, in Fourier domain, we have:

$$\begin{aligned}\mathscr{F}\{I_{\text{slopes}}(\mathbf{r})\} &= \frac{1}{2}\mathscr{F}\left\{\exp\left[\mathrm{j}\left(\omega x-\frac{\lambda z}{2\pi}\omega\nabla_x\phi\right)\right]+\exp\left[-\mathrm{j}\left(\omega x-\frac{\lambda z}{2\pi}\omega\nabla_x\phi\right)\right]\right\}+\\&\quad\frac{1}{2}\mathscr{F}\left\{\exp\left[\mathrm{j}\left(\omega y-\frac{\lambda z}{2\pi}\omega\nabla_y\phi\right)\right]+\exp\left[-\mathrm{j}\left(\omega y-\frac{\lambda z}{2\pi}\omega\nabla_y\phi\right)\right]\right\}\\&=\frac{1}{2}\left\{D_x\left(\boldsymbol{\rho}_x-\frac{\omega}{2\pi},\boldsymbol{\rho}_y\right)+\overline{D}_x\left(\boldsymbol{\rho}_x+\frac{\omega}{2\pi},\boldsymbol{\rho}_y\right)+D_y\left(\boldsymbol{\rho}_x,\boldsymbol{\rho}_y-\frac{\omega}{2\pi}\right)+\overline{D}_y\left(\boldsymbol{\rho}_x,\boldsymbol{\rho}_y+\frac{\omega}{2\pi}\right)\right\},\end{aligned} \tag{S.32}$$

where $\bar{\cdot}$ denotes complex conjugate, and $D_x(\boldsymbol{\rho})$ and $D_y(\boldsymbol{\rho})$ are the Fourier transforms of $d_x(\mathbf{r})$ and $d_y(\mathbf{r})$ respectively, where $d_x(\mathbf{r})$ and $d_y(\mathbf{r})$ are defined as:

$$d_x(\mathbf{r}) = -\frac{\lambda z}{2\pi}\omega\nabla_x\phi \qquad \text{and} \qquad d_y(\mathbf{r}) = -\frac{\lambda z}{2\pi}\omega\nabla_y\phi. \tag{S.33}$$

This permits one to selectively filter only the spectrum components $D_x(\boldsymbol{\rho}_x-\frac{\omega}{2\pi},\boldsymbol{\rho}_y)$ and $D_y(\boldsymbol{\rho}_x,\boldsymbol{\rho}_y-\frac{\omega}{2\pi})$ on the carrier frequency ω, shift it down to the origin to remove its carrier frequency, and obtain $D_x(\boldsymbol{\rho})$ and $D_y(\boldsymbol{\rho})$, which are the spectrums of $d_x(\mathbf{r})$ and $d_y(\mathbf{r})$, i.e. the linearly scaled wavefront slopes. A Poisson integration is then imposed on the previously obtained wavefront slopes to obtain the desired phase $\phi(\mathbf{r})$. As a result, with a specifically designed mask, lateral shearing interferometers are able to resolve phase information in Fourier domain directly, thus a reference image is not necessary to be taken prior to phase measurements.

Amplitude-contained flow-tracking model

If drop out only the wavefront curvature term, from Eq. (S.20) we have:

$$I\left(\mathbf{r}+\frac{\lambda z}{2\pi}\nabla\phi\right) = |A(\mathbf{r})|^2 I_0(\mathbf{r}), \tag{S.34}$$

which we recognize as the models typically employed in X-ray phase imaging applications.[4] Another commonly seen formulation is:

$$I(\mathbf{r}) = |A(\mathbf{r})|^2 I_0\left(\mathbf{r}-\frac{\lambda z}{2\pi}\nabla\phi\right), \tag{S.35}$$

which can be derived by ignoring the sample scalar field $f_0(\mathbf{r})$ diffraction and approximate $f_z(\mathbf{r}) \approx f_0(\mathbf{r}) = A(\mathbf{r})\exp[\mathrm{j}\phi(\mathbf{r})]$ in Eq. (S.15). Lateral shearing interferometers are still a special case where simultaneous amplitude and phase reconstruction are obtained in Fourier domain[27] as reviewed below. For simplicity we consider again the grating $p(\mathbf{r}) = \cos(\omega x/2)\cos(\omega y/2)$ while other gratings can be derived in a similar way. Because of the amplitude term, the received image becomes:

$$I(\mathbf{r}) \underset{\text{(S.31)}}{\overset{\text{(S.35)}}{=}} \underbrace{|A(\mathbf{r})|^2}_{\text{DC term}} - \underbrace{|A(\mathbf{r})|^2\left(\cos\left[\omega\left(x-\frac{\lambda z}{2\pi}\nabla_x\phi\right)\right]+\cos\left[\omega\left(y-\frac{\lambda z}{2\pi}\nabla_y\phi\right)\right]\right)}_{I_{\text{slopes}}(\mathbf{r})\text{, the wavefront slopes encoded terms}} + \cdots, \tag{S.36}$$

where we neglect higher tangled terms including the multiplication parts between x and y. In Fourier domain:

$$\mathscr{F}\{I(\mathbf{r})\} \overset{\text{(S.36)}}{\approx} \mathscr{A}(\boldsymbol{\rho}) - \mathscr{A}(\boldsymbol{\rho}) * \mathscr{F}\{I_{\text{slopes}}(\mathbf{r})\}, \tag{S.37}$$

where $\mathscr{A}(\boldsymbol{\rho})$ denotes the Fourier transform of $|A(\mathbf{r})|^2$, and $*$ denotes convolution. Now the central low frequency spectrum encodes the sample amplitude spectrum $\mathscr{A}(\boldsymbol{\rho})$, and can be extracted first to detangle for obtaining the wavefront slopes spectrum $\mathscr{F}\{I_{\text{slopes}}(\mathbf{r})\}$ from $\mathscr{F}\{I(\mathbf{r})\}$. Note the numerical reconstruction is in Fourier domain, and can be potentially improved by the dual approach in spatial domain.[32]

Comparison with Zanette et al.'s model

Finally note the model in Zanette et al.[5], which modifies Eq. (S.35) by incorporating a dark field $D(\mathbf{r})$ that represents contribution from higher order phase e.g. from $\nabla^2\phi$:

$$I(\mathbf{r}) = |A(\mathbf{r})|^2\left[\bar{I}_0 + D(\mathbf{r})\Delta I_0\left(\mathbf{r}-\frac{\lambda z}{2\pi}\nabla\phi\right)\right], \tag{S.38}$$

where $\bar{I}_0$ is the mean value of $I_0(\mathbf{r})$, and $\Delta I_0(\mathbf{r}) = I_0(\mathbf{r}) - \bar{I}_0$. With Eq. (S.20) we know part of their $D(\mathbf{r})$ origins from the caustic term $1 - \lambda z \nabla^2 \phi / (2\pi)$ that contains wavefront curvature $\nabla^2 \phi$, representing sample diffraction under a distorted wavefront, which is part of refraction. Therefore, the dark field $D(\mathbf{r})$ in Eq. (S.38) can be further refined to contain only scattering terms. Eq. (S.20) provides new insights for incorporating dark field term into the image formation model.

Decoupling amplitude, phase, and caustic effects

Each contribution term in the main result Eq. (S.20) can be interpreted as following:

$$\underbrace{I\left(\mathbf{r} + \frac{\lambda z}{2\pi}\nabla\phi\right)}_{\text{measurement}} = \underbrace{|A(\mathbf{r})|^2}_{\text{amplitude}} \times \underbrace{\left(1 - \frac{\lambda z}{2\pi}\nabla^2\phi\right)}_{\text{caustic by defocusing}} \times \underbrace{I_0(\mathbf{r})}_{\text{reference}} \tag{S.39}$$

Notice above formula enables the separation between sample amplitude and caustic effects caused by defocusing. To recall, our task is, knowing a nominal wavelength λ and a pre-calibrated z, given the image pair $I_0(\mathbf{r})$ and $I(\mathbf{r})$, solve for sample amplitude $A(\mathbf{r})$ due to absorption, and solve for phase $\phi(\mathbf{r})$ due to sample shape refraction, at the same time. To do this, we devise an alternating optimization scheme, i.e. updating amplitude and phase in alternation. For the amplitude update, since the contribution from defocusing caused by local wavefront curvature $\nabla^2\phi$ is usually small, we absorb the caustic term into amplitude as unknown. Consequently, the amplitude estimation reduces to be a variant of the classical Rudin-Osher-Fatemi (ROF) denoising problem[33], which can be efficiently solved by modern splitting algorithms. Given the previous amplitude estimation, the phase update simplifies to an optical flow problem, which is solved by a variant of solver in Wang et al.[7] with new priors for microscopy images. Until convergence, deducing the caustic term from the estimated intensity term. To summarize, the final problem is, solve a quasi amplitude term $\tilde{A}(\mathbf{r})$ and phase $\phi(\mathbf{r})$ from reference $I_0(\mathbf{r})$ and measurement $I(\mathbf{r})$ based on:

$$I\left(\mathbf{r} + \frac{\lambda z}{2\pi}\nabla\phi\right) = |\tilde{A}(\mathbf{r})|^2 I_0(\mathbf{r}). \tag{S.40}$$

After obtaining $\tilde{A}(\mathbf{r})$ and $\phi(\mathbf{r})$, the pure amplitude term is calculated as:

$$A(\mathbf{r}) = \tilde{A}(\mathbf{r})\sqrt{1 + \frac{\lambda z}{2\pi}\nabla^2\phi}. \tag{S.41}$$

Regularization and proper optimization techniques improve solution plausibility, and will be discussed in Section S.2 where optimization frameworks and efficient numerical algorithms are proposed to solve above equations in a numerical manner.

S.2 Optimization

We now discuss how to discretize and solve Eq. (S.40) in a numerical manner, which involves solving an optimization problem in terms of linear algebra. In the following vectors and matrices are denoted as bold small and capital letters, respectively.

Absorbing $\lambda z/(2\pi)$ into phase and discretize Eq. (S.40) yields following joint optimization problem:

$$\underset{\mathbf{a},\boldsymbol{\phi}}{\text{minimize}} \quad \|\boldsymbol{i}(\mathbf{r} + \nabla\boldsymbol{\phi}) - \mathbf{a} \odot \boldsymbol{i}_0(\mathbf{r})\|_2^2 + \underbrace{\alpha\|\nabla\boldsymbol{\phi}\|_1 + \beta\|\mathbf{K}\boldsymbol{\phi}\|_2^2}_{\Gamma_{\text{phase}}(\boldsymbol{\phi})} + \underbrace{\gamma\|\mathbf{K}\mathbf{a}\|_1 + \tau\|\mathbf{K}\mathbf{a}\|_2^2}_{\Gamma_{\text{intensity}}(\mathbf{a})}, \tag{S.42}$$

where $\odot$ denotes Hadamard product, $\mathbf{K} = \begin{bmatrix} \nabla \\ \nabla^2 \end{bmatrix}$ is a concatenated matrix of gradient and Laplacian operators, $\mathbf{a}$ and $\boldsymbol{\phi}$ are the intensity (including the caustic effect) and phase information that we would like to recover. Equation (S.42) is a non-convex problem and is highly ill-posed. To see this, let total pixel numbers be N, note we have $2N$ unknowns (intensity and phase) to estimate whereas only given N equations from Eq. (S.20). The two regularizers, phase prior $\Gamma_{\text{phase}}(\boldsymbol{\phi})$ and intensity prior $\Gamma_{\text{intensity}}(\mathbf{a})$ are hence introduced here to help reducing the ill-posed, but the estimation will still be hard.

To tackle this problem, we devise an alternating algorithm to alternatively solve for intensity and phase, i.e. solve for one when given the other is fixed. Algorithm S.1 shows this procedure. In practice we found just a few alternating iterations (< 5) are sufficient for a satisfactory convergence. We now discuss each updating step in more details.

Intensity update

We recognize the intensity update step as a variant of the classical ROF denoising problem:[33]

$$\underset{\mathbf{a}}{\text{minimize}} \quad \|\mathbf{a} \odot \boldsymbol{i}_0(\mathbf{r}) - \boldsymbol{i}(\mathbf{r} + \nabla\boldsymbol{\phi}^K)\|_2^2 + \gamma\|\nabla\mathbf{a}\|_1 + \tau\|\mathbf{K}\mathbf{a}\|_2^2. \tag{S.43}$$

Algorithm S.1 Alternating intensity and phase estimation for Eq. (S.42).

Initialize $\mathbf{a}^0 = \mathbf{1}$ and $\boldsymbol{\phi}^0 = \mathbf{0}$;
while not converge **do**
  $\mathbf{a}^{K+1} = \arg\min_{\mathbf{a}} \quad \left\| \boldsymbol{i}(\mathbf{r} + \nabla \boldsymbol{\phi}^K) - \mathbf{a} \odot \boldsymbol{i}_0(\mathbf{r}) \right\|_2^2 + \Gamma_{\text{intensity}}(\mathbf{a})$;   ▷ Intensity update: solve **a** given $\boldsymbol{\phi}$
  $\boldsymbol{\phi}^{K+1} = \arg\min_{\boldsymbol{\phi}} \quad \left\| \boldsymbol{i}(\mathbf{r} + \nabla \boldsymbol{\phi}) - \mathbf{a}^{K+1} \odot \boldsymbol{i}_0(\mathbf{r}) \right\|_2^2 + \Gamma_{\text{phase}}(\boldsymbol{\phi})$;   ▷ Phase update: solve $\boldsymbol{\phi}$ given **a**
end while

Equation (S.43) is convex but non-differentiable. By introducing a slack variable $\mathbf{b} = \nabla \mathbf{a}$ that represents image gradient, via de-coupling diagonalization (though not strictly equivalent, in practice we found in this formation it is easier to formulate the solver and to converge), denoting $\boldsymbol{i}_{\text{warp}}^K = \boldsymbol{i}(\mathbf{r} + \nabla \boldsymbol{\phi}^K)/\boldsymbol{i}_0(\mathbf{r})$, the original objective function in Eq. (S.43) can be split and approximated as:

$$\begin{aligned} \underset{\mathbf{a},\mathbf{b}}{\text{minimize}} \quad & \underbrace{\left\| \mathbf{a} - \boldsymbol{i}_{\text{warp}}^K \right\|_2^2 + \tau_{\text{new}} \left\| \mathbf{K}\mathbf{a} \right\|_2^2}_{f(\mathbf{a})} + \underbrace{\gamma_{\text{new}} \left\| \mathbf{b} \right\|_1}_{g(\mathbf{b})}, \\ \text{subject to} \quad & \mathbf{b} = \nabla \mathbf{a}, \end{aligned} \tag{S.44}$$

where $\tau_{\text{new}} = \tau / \overline{(\boldsymbol{i}_{\text{warp}}^K)^2}$ and $\gamma_{\text{new}} = \gamma / \overline{(\boldsymbol{i}_{\text{warp}}^K)^2}$ where over-line denotes the mean. Apply the Alternating Direction Method of Multipliers (ADMM) method[34] to Eq. (S.44), we yield Algorithm S.2, where $\mathbf{prox}_{g/\mu}(\mathbf{u})$ denotes the proximal operator[35] of function g with parameter μ, given the input vector $\mathbf{u}$. And $\boldsymbol{\eta}$ is the dual variable. Now we briefly discuss each updating step.

Algorithm S.2 ADMM for solving Eq. (S.44).

Initialize $\mathbf{a}^0$, $\mathbf{b}^0$ and $\boldsymbol{\eta}^0$, set $\mu > 0$;
while not converge **do**
  $\mathbf{a}^{k+1} \leftarrow \arg\min_{\mathbf{a}} \quad f(\mathbf{a}) + \mu \left\| \nabla \mathbf{a} - \mathbf{b}^k + \boldsymbol{\eta}^k \right\|_2^2$;   ▷ **a**-update
  $\mathbf{b}^{k+1} \leftarrow \mathbf{prox}_{g/\mu}(\nabla \mathbf{a}^{k+1} + \boldsymbol{\eta}^k)$;   ▷ **b**-update
  $\boldsymbol{\eta}^{k+1} \leftarrow \boldsymbol{\eta}^k + \nabla \mathbf{a}^{k+1} - \mathbf{b}^{k+1}$;   ▷ $\boldsymbol{\eta}$-update
end while

a-update. This step implements a straightforward Poisson solver, and we solve it in the spectral domain assuming symmetric boundary conditions. Exploiting the Discrete Cosine Transforms (DCT), denoted as $\mathscr{F}_{\text{DCT}}$, then:

$$\begin{aligned} \mathbf{a}^{k+1} &= \arg\min_{\mathbf{a}} \quad \left\| \mathbf{a} - \boldsymbol{i}_{\text{warp}}^K \right\|_2^2 + \tau_{\text{new}} \left\| \mathbf{K}\mathbf{a} \right\|_2^2 + \mu \left\| \nabla \mathbf{a} - \mathbf{b}^k + \boldsymbol{\zeta}^k \right\|_2^2 \\ &= \left(\mathbf{I} + \tau_{\text{new}} \mathbf{K}^{\mathrm{T}} \mathbf{K} + \mu \nabla^2 \right)^{-1} \left(\boldsymbol{i}_{\text{warp}}^K + \mu \nabla^{\mathrm{T}} (\mathbf{b}^k - \boldsymbol{\zeta}^k) \right) \\ &= \mathscr{F}_{\text{DCT}}^{-1} \left(\frac{\boldsymbol{i}_{\text{warp}}^K + \mu \mathscr{F}_{\text{DCT}} \left(\nabla^{\mathrm{T}} (\mathbf{b}^k - \boldsymbol{\zeta}^k) \right)}{1 + (\mu + \tau_{\text{new}}) \mathscr{F}_{\text{DCT}}(\nabla^2) + \tau_{\text{new}} \mathscr{F}_{\text{DCT}}(\nabla^4)} \right). \end{aligned} \tag{S.45}$$

Exploiting the Fast Fourier Transforms (FFT) algorithms for all the DCT operations, the **a**-update can be efficiently done in parallel.

b-update. This step is an element-wise estimation and the solution is readily obtained by the so-called shrinkage operator that is embarrassingly parallel, where $\text{sign}(\cdot)$ denotes the element-wise signum function:

$$\begin{aligned} \mathbf{b}^{k+1} &= \mathbf{prox}_{g/\mu}(\nabla \mathbf{a}^{k+1} + \boldsymbol{\eta}^k) \\ &= \arg\min_{\mathbf{b}} \quad \gamma_{\text{new}} \left\| \mathbf{b} \right\|_1 + \mu \left\| \mathbf{b} - (\nabla \mathbf{a}^{k+1} + \boldsymbol{\eta}^k) \right\|_2^2 \\ &= \text{sign}\left(\nabla \mathbf{a}^{k+1} + \boldsymbol{\eta}^k \right) \cdot \max\left(\left| \nabla \mathbf{a}^{k+1} + \boldsymbol{\eta}^k \right| - \frac{\gamma_{\text{new}}}{2\mu}, \mathbf{0} \right). \end{aligned} \tag{S.46}$$

After obtaining $\mathbf{a}^K$, a median filter (window of 3×3) is imposed to further suppress speckle noise. In practice we found the median filtering is significant for better performance.

Phase update

For phase update, people usually do the digital image correlation method[36], which however in computer vision is known as a variant of the famous Lucas–Kanade method[29] for optical flow computation. Here we modify and improve our previously proposed wavefront solver to fit the assumption for normal microscopy samples. Consequently the wavefront solver presented here is a variant of Wang et al.[7] Recall the following optimization problem to update phase:

$$\underset{\boldsymbol{\phi}}{\text{minimize}} \quad \left\| \boldsymbol{i}(\mathbf{r}+\nabla\boldsymbol{\phi}) - \mathbf{a}^{K+1} \odot \boldsymbol{i}_0(\mathbf{r}) \right\|_2^2 + \alpha \left\| \nabla\boldsymbol{\phi} \right\|_1 + \beta \left\| \mathbf{K}\boldsymbol{\phi} \right\|_2^2, \tag{S.47}$$

where $\alpha > 0$ and $\beta > 0$ are weighting parameters. Note the data fitting term (ℓ_2-norm) is non-convex and one of the data prior term (ℓ_1-norm) is convex but non-smooth and non-differentiable. Since the phase shifts are usually small, we linearize $\boldsymbol{i}(\mathbf{r}+\nabla\boldsymbol{\phi})$ around $\mathbf{r}$. It yields:

$$\underset{\boldsymbol{\phi}}{\text{minimize}} \quad \left\| \nabla\boldsymbol{i} \cdot \nabla\boldsymbol{\phi} + \boldsymbol{i}(\mathbf{r}) - \mathbf{a}^{K+1} \odot \boldsymbol{i}_0(\mathbf{r}) \right\|_2^2 + \alpha \left\| \nabla\boldsymbol{\phi} \right\|_1 + \beta \left\| \mathbf{K}\boldsymbol{\phi} \right\|_2^2. \tag{S.48}$$

To handle the boundary condition (which may introduce reconstruction artifacts in conventional phase-from-slope techniques), we add a selection matrix $\mathbf{M}$ to include the unknown boundary values as additional variables to be optimized.[37] In linear algebra, denote $\mathbf{g}_t = \boldsymbol{i}(\mathbf{r}) - \mathbf{a}^{K+1} \odot \boldsymbol{i}_0(\mathbf{r})$, Eq. (S.48) reads as:

$$\underset{\boldsymbol{\phi}}{\text{minimize}} \quad \left\| \nabla\boldsymbol{i} \cdot \mathbf{M}\nabla\boldsymbol{\phi} + \mathbf{g}_t \right\|_2^2 + \alpha \left\| \nabla\boldsymbol{\phi} \right\|_1 + \beta \left\| \mathbf{K}\boldsymbol{\phi} \right\|_2^2. \tag{S.49}$$

Equation (S.49) is convex but non-differentiable. By introducing a slack variable $\mathbf{w} = \nabla\boldsymbol{\phi}$ that represents phase gradient, the original objective function in Eq. (S.49) can be split as:

$$\begin{aligned} \underset{\boldsymbol{\phi},\mathbf{w}}{\text{minimize}} \quad & \underbrace{\beta \left\| \mathbf{K}\boldsymbol{\phi} \right\|_2^2}_{f(\boldsymbol{\phi})} + \underbrace{\left\| \nabla\boldsymbol{i} \cdot \mathbf{M}\mathbf{w} + \mathbf{g}_t \right\|_2^2 + \alpha \left\| \mathbf{w} \right\|_1}_{g(\mathbf{w})}, \\ \text{subject to} \quad & \mathbf{w} = \nabla\boldsymbol{\phi}. \end{aligned} \tag{S.50}$$

Apply the Alternating Direction Method of Multipliers (ADMM) method[34] to Eq. (S.50), we yield Algorithm S.3.

Algorithm S.3 ADMM for solving Eq. (S.50).

Initialize $\boldsymbol{\phi}^0$, $\mathbf{w}^0$ and $\boldsymbol{\eta}^0$, set $\mu > 0$;
while not converge **do**
    $\boldsymbol{\phi}^{k+1} \leftarrow \underset{\boldsymbol{\phi}}{\arg\min} \quad f(\boldsymbol{\phi}) + \mu \left\| \nabla\boldsymbol{\phi} - \mathbf{w}^k + \boldsymbol{\eta}^k \right\|_2^2$; ▷ $\boldsymbol{\phi}$-update
    $\mathbf{w}^{k+1} \leftarrow \mathbf{prox}_{g/\mu}(\nabla\boldsymbol{\phi}^{k+1} + \boldsymbol{\eta}^k)$; ▷ $\mathbf{w}$-update
    $\boldsymbol{\eta}^{k+1} \leftarrow \boldsymbol{\eta}^k + \nabla\boldsymbol{\phi}^{k+1} - \mathbf{w}^{k+1}$; ▷ $\boldsymbol{\eta}$-update
end while

$\boldsymbol{\phi}$-update. This step is a Poisson solver, and we solve it in the spectrum domain assuming symmetric boundary conditions:

$$\begin{aligned} \boldsymbol{\phi}^{k+1} &= \underset{\boldsymbol{\phi}}{\arg\min} \quad \beta \left\| \mathbf{K}\boldsymbol{\phi} \right\|_2^2 + \mu \left\| \nabla\boldsymbol{\phi} - \mathbf{w}^k + \boldsymbol{\zeta}^k \right\|_2^2 \\ &= \left(\beta \mathbf{K}^{\mathrm{T}}\mathbf{K} + \mu \nabla^2 \right)^{-1} \mu \nabla^{\mathrm{T}} (\mathbf{w}^k - \boldsymbol{\zeta}^k) \\ &= \mathscr{F}_{\mathrm{DCT}}^{-1} \left(\frac{\mu \mathscr{F}_{\mathrm{DCT}} \left(\nabla^{\mathrm{T}} (\mathbf{w}^k - \boldsymbol{\zeta}^k) \right)}{\beta \mathscr{F}_{\mathrm{DCT}}(\nabla^4) + (\beta + \mu) \mathscr{F}_{\mathrm{DCT}}(\nabla^2)} \right). \end{aligned} \tag{S.51}$$

w-update. This involves evaluation of $\mathbf{w}^{k+1} = \mathbf{prox}_{g/\mu}(\mathbf{u})$ with $\mathbf{u} = \nabla\boldsymbol{\phi}^{k+1} + \boldsymbol{\eta}^k$, i.e. the proximal operator[35] of $g(\mathbf{w})$ with parameter μ, which is defined as:

$$\begin{aligned} \mathbf{prox}_{g/\mu}(\mathbf{u}) &= \underset{\mathbf{w}}{\arg\min} \quad g(\mathbf{w}) + \mu \left\| \mathbf{w} - \mathbf{u} \right\|_2^2 \\ &= \underset{\mathbf{w}}{\arg\min} \quad \left\| \nabla\boldsymbol{i} \cdot \mathbf{M}\mathbf{w} + \mathbf{g}_t \right\|_2^2 + \mu \left\| \mathbf{w} - \mathbf{u} \right\|_2^2 + \alpha \left\| \mathbf{w} \right\|_1. \end{aligned} \tag{S.52}$$

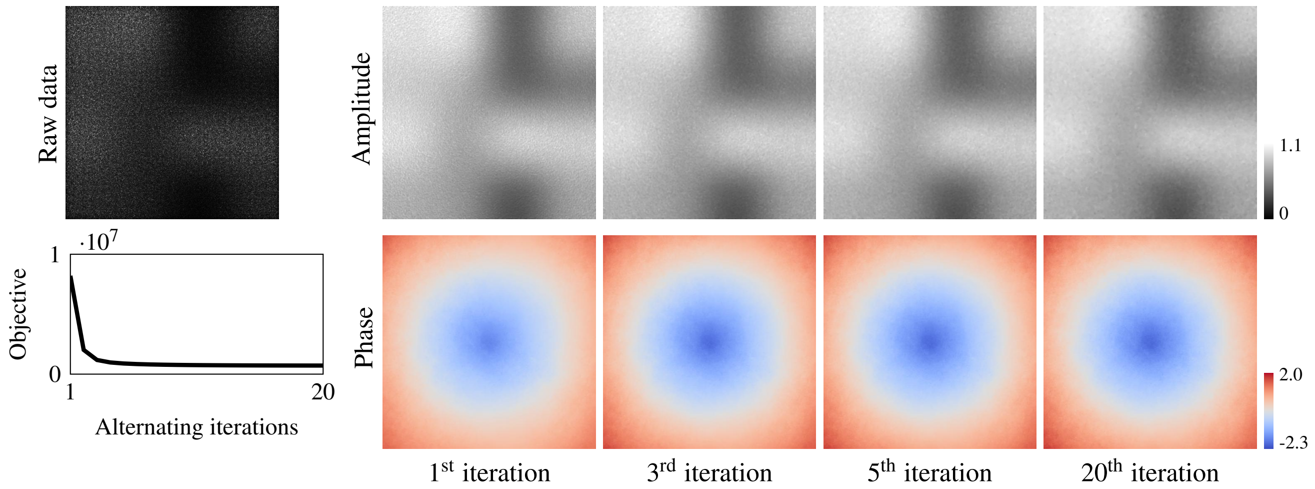

Figure S.2. Solver convergence. Our solver converges in a few iterations. In practice 3 alternating iterations are sufficient for convergence. Phase units are µm.

Equation (S.52) is separable in $\mathbb{R}^2$ as many Least-Absolute-Shrinkage-and Selection-Operator (LASSO) problems, which can be solved in the dual form in the formations of linear systems, which are 2-by-2 small matrices that have closed-form solutions for inversion. Conclusively, for $\mathbf{w}$-update we have closed-form solutions, which are all element-wise operations and hence the $\mathbf{w}$-update step is easy parallelization as well. By far, all the operations in Algorithm S.3 can be parallelized, and hence the total computation can be efficiently run on distributed computing machines. e.g. on a commercially affordable GPU.

After obtaining $\boldsymbol{\phi}^K$, we approximate the warped measurement image as $\boldsymbol{i}(\mathbf{r}+\nabla\boldsymbol{\phi}^K) \approx \boldsymbol{i}(\mathbf{r}) + \nabla\boldsymbol{i}\cdot\nabla\boldsymbol{\phi}^K$, and run next intensity update estimation in Algorithm S.3. For even larger wavefronts, pyramid schemes can be adopted, as in Wang et al.[38] The spirit behind is a similar manner of the nonlinear warping scheme in optical flow.[39]

S.3 Additional Results

In this section additional results are presented.

Solver performance

In Fig. S.2 we show solver convergence in terms of alternating iterations. The two inner loops (amplitude-update) and (phase-update) are both 10 iterations. Our solver converges fast in a few iterations. In practice we chose 3 alternating iterations.

Comparison with classical solver

Here we do a numerical comparison between classical algorithm and our proposed one, using synthetic data.

Classical solver implementation

Recall that classical speckle-pattern tracking algorithms[1,2,6,8,22,23] are able to reconstruct simultaneously (in our notation): a bright field image $|A(\mathbf{r})|^2$, a phase image $\phi(\mathbf{r})$, and a dark-field image $D(\mathbf{r})$ from a single raw measurement speckle-pattern image $I(\mathbf{r})$ and a pre-calibrated reference image $I_0(\mathbf{r})$. For a fair comparison, we implement the baseline classical speckle-pattern tracking algorithm as following:

Bright field image. The bright field image is computed as:[6]

$$|A(\mathbf{r})|^2 \approx \frac{\mu_w\big(I(\mathbf{r})\big)}{\mu_w\big(I_0(\mathbf{r})\big)}, \tag{S.53}$$

where $\mu_w(\cdot)$ denotes mean operation inside window w. The window size of w is chosen to be 3×3 to preserve maximum details while suppressing noise at the same time. In practice we found a median filtering on $|A(\mathbf{r})|^2$ decrease the phase image accuracy, so there is no post-processing after obtaining the bright field image.

Phase image. To reconstruct the phase shifts, classical algorithm tracks the image pair $I(\mathbf{r})$ and $|A(\mathbf{r})|^2 I_0(\mathbf{r})$ using digital image correlation (DIC) method[36] to get the wavefront slopes $w(\mathbf{r})$, and then integrate the slopes to get the final wavefront

$\phi(\mathbf{r})$. Here we employ the `imregdemons` MATLAB function as suggested by Berto et al.[8] to compute the slopes $w(\mathbf{r})$, and then apply a Poisson solver (with pre-zero-paddings and symmetric boundary condition assumption) to obtain the final phase image. In math:

$$\phi(\mathbf{r}) = \mathscr{F}_{\mathrm{DCT}}^{-1}\left(\frac{\mathscr{F}_{\mathrm{DCT}}\left(\nabla^{\mathrm{T}} w(\mathbf{r})\right)}{\mathscr{F}_{\mathrm{DCT}}(\nabla^2)}\right). \tag{S.54}$$

Note this approach could suppress the phase reconstruction: instead of solving for $\phi \in \mathbb{R}^N$ directly, it solves for an intermediate wavefront slopes $\nabla\phi \in \mathbb{R}^{2N}$, and then integrate to space $\mathbb{R}^N$, during which possible accuracy loss could happen.

Comparison using synthetic data

Multiple 512×512 size diffraction patterns are simulated by the angular spectrum method with filtering.[11,40] The sensor and mask pixel sizes are set as 6.45 µm, and the distance between mask and sensor is 1.5 mm. Wavelength is set as 550 nm. Reconstruction comparison between classical algorithm and ours are shown in Fig. S.3 (amplitude) and Fig. S.4 (phase), with the absolute error shown and mean value calculated as well. For amplitude reconstruction, both algorithms have the difficulty reconstructing the sharp edges, but for smooth amplitude changes the reconstructions are successful, and the differences between the two algorithms are subtle. However, for phase reconstruction, specifically for the spherical wavefront and the lenslets wavefront (large distorted wavefronts at the present of local curvature), reconstruction error of our algorithm is smaller than the classical one, which underestimates the wavefront distortion. This is probably due to the fact that, from the N given equations (i.e. the measurement image), classical algorithm suffers an accuracy loss when first solving the intermediate wavefront slopes (of size $2N$) and then integrate to get the final phase (of size N), while our algorithm solves for the phase (of size N) directly from the given N equations. But the difference between the two is not significant.

Model evaluation

It is also possible to compute a pseudo dark field image from the image pair $I_0(\mathbf{r})$ and $I(\mathbf{r})$ as following.

Dark field image. The dark field image is related to the decrease of speckle-pattern contrast. It can be computed as:[6]

$$D(\mathbf{r}) \approx \frac{\sigma_w\left(I(\mathbf{r} + \frac{\lambda z}{2\pi}\nabla\phi)\right)}{\sigma_w\left(|A(\mathbf{r})|^2 I_0(\mathbf{r})\right)}, \tag{S.55}$$

where $\sigma_w(\cdot)$ denotes standard deviation operation inside window w. The window size of w is chosen to be 3×3. Fig. S.5 shows one example of the construction of amplitude $A(\mathbf{r})$, phase $\phi(\mathbf{r})$, and dark field scattering image $D(\mathbf{r})$ from image pair $I_0(\mathbf{r})$ and $I(\mathbf{r})$, as well as the caustic term computed from Eq. (S.20). As a result, the scattering image is very poor, while the local wavefront curvature is small that is negligible by the tracking model. To the contrary, for visible light microscopy applications, in TIE imaging the wavefront Laplacian term is significant because the usual distance between defocusing intensity measurements ($z \approx 20\,\mu$m) are much smaller than that in speckle-pattern tracking techniques ($z \approx 1.5\,$mm).

Additional experimental reconstructions

In Fig. S.6 we show further reconstruction results on laboratory-acquired raw data.

Appendix

Fourier transform of a complex Gaussian function

Given value $a \in \mathbb{R}$, the Fourier transform of $f(\mathbf{r}) = \exp\left(\mathrm{j}a\|\mathbf{r}\|_2^2\right)$ is, in real dual domain $\boldsymbol{\rho}$:

$$\begin{aligned} F(\boldsymbol{\rho}) &= \int \exp(-\mathrm{j}\boldsymbol{\rho}\cdot\mathbf{r}) \exp\left(\mathrm{j}a\|\mathbf{r}\|_2^2\right) \mathrm{d}\mathbf{r} \\ &= \frac{1}{\sqrt{\pi a}} \exp\left(\mathrm{j}\frac{\pi - \frac{1}{a}\|\boldsymbol{\rho}\|_2^2}{4}\right). \end{aligned} \tag{S.56}$$

There is no convergence guarantee for this integral in mathematics, but in engineering this analytical expression is sufficient to support our analysis in this Supplementary.

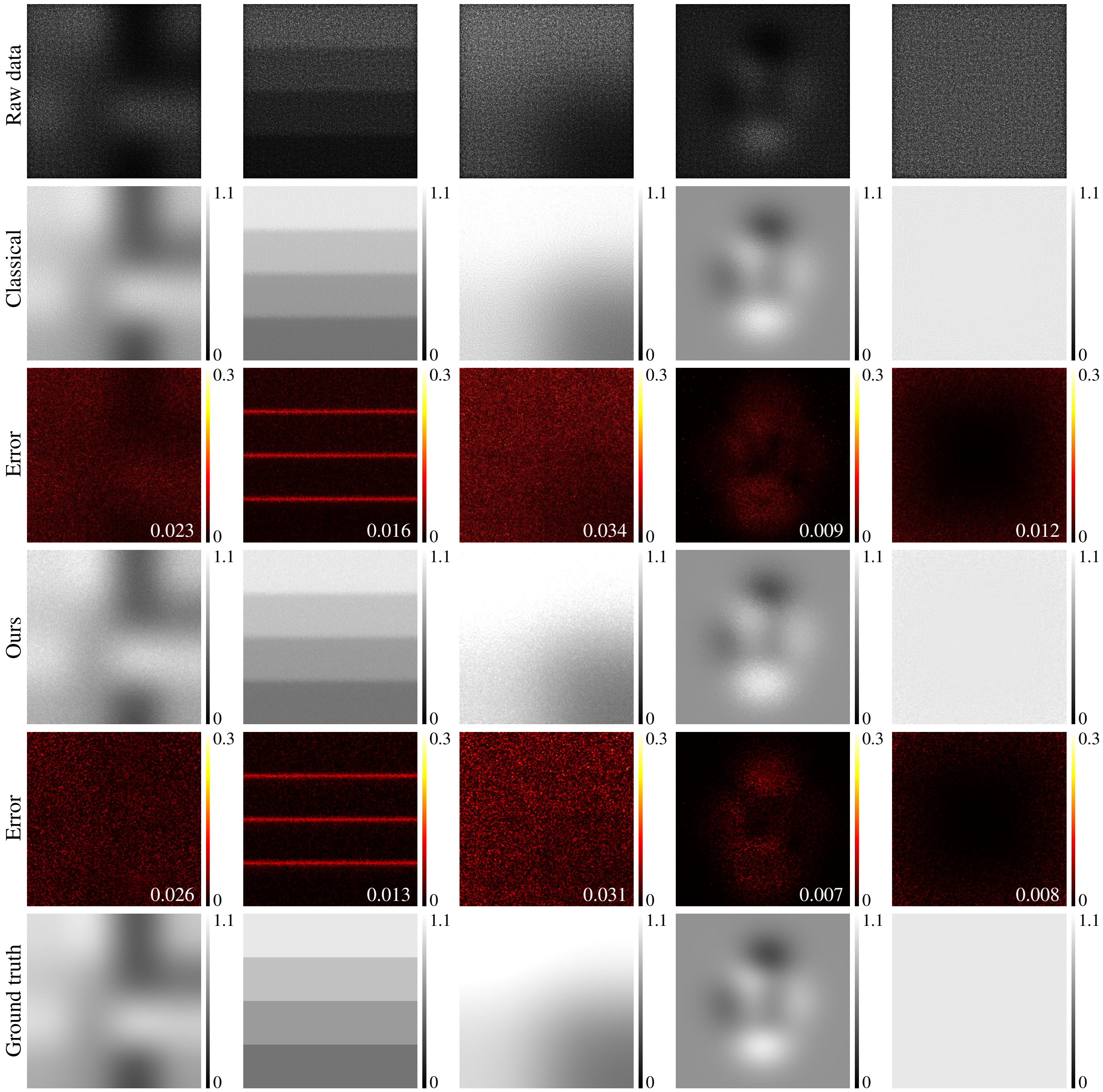

Figure S.3. Synthetic comparison between classical speckle-tracking algorithm and ours (amplitude). Mean absolute error is shown at the corners.

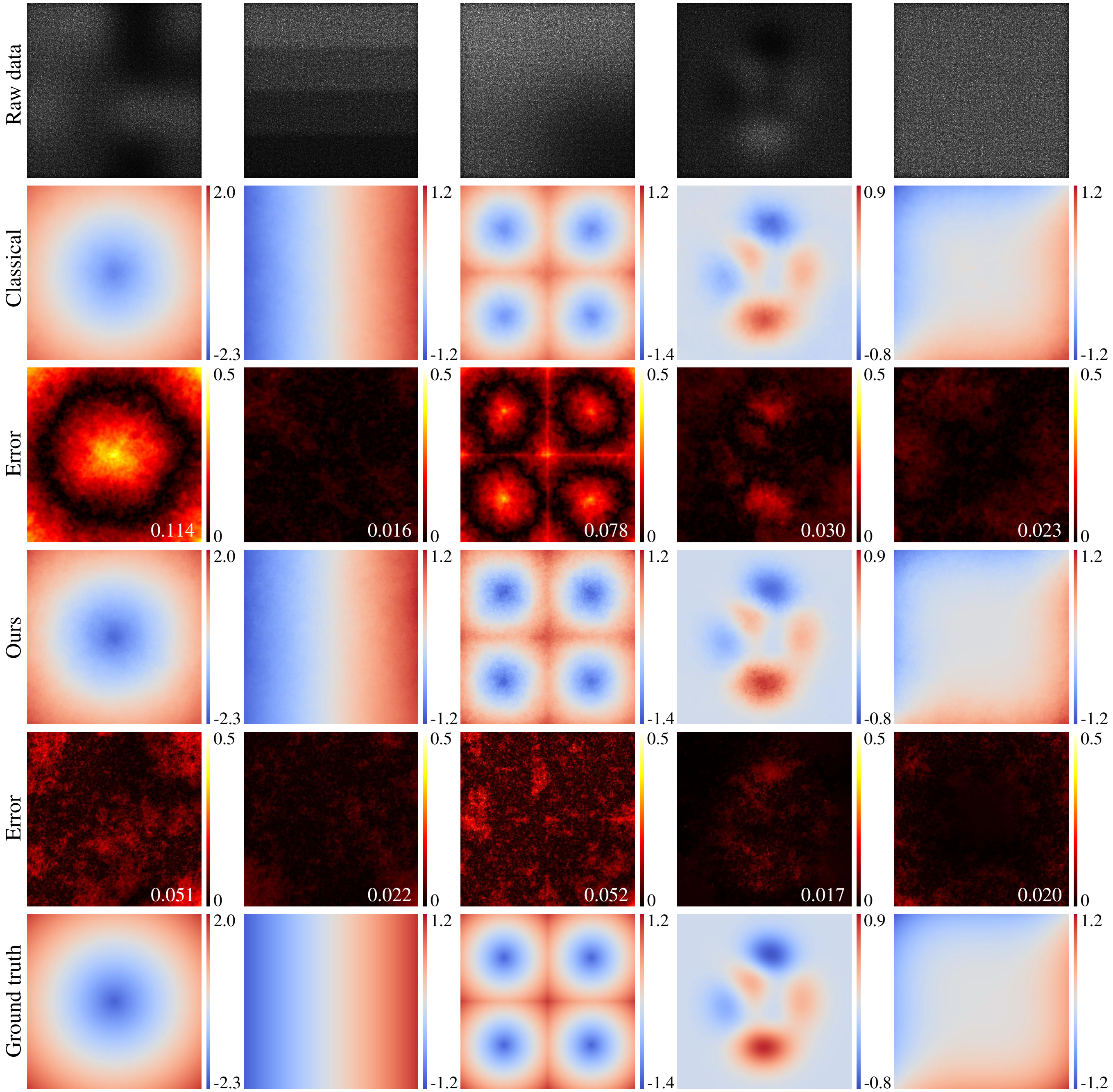

Figure S.4. Synthetic comparison between classical speckle-tracking algorithm and ours (phase). Mean absolute error is shown at the corners. Units are μm.

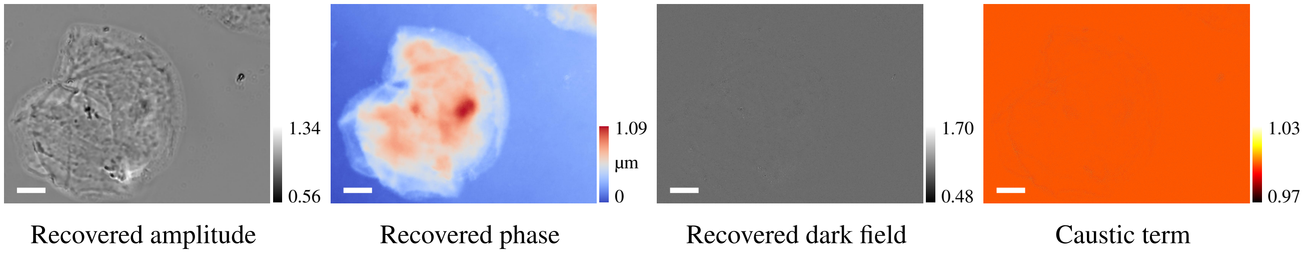

Figure S.5. A full reconstruction example from experimental data, where amplitude, phase, dark field, and caustic term are recovered from the raw speckle image data. The dark field image is not obvious, and the caustic effect is small enough to be neglected as been done in most other speckle-pattern tracking techniques. Scale bars are 10 μm.

Computational caustic image formulation

To be consistent with the paper, here we replicate the derivation from Damberg and Heidrich[12] for referencing Eq. (S.22). Let $\mathbf{r}$ and $\mathbf{r}'$ be the coordinates at the mask plane and sensor plane respectively, for one single ray, by geometry at wavelength λ:

$$\mathbf{r}' = \mathbf{r} + z\frac{\lambda}{2\pi}\nabla\phi(\mathbf{r}), \tag{S.57}$$

where we employ the small angle approximation that $\sin\theta \approx \theta$ when $\frac{\lambda}{2\pi}\nabla\phi(\mathbf{r}) = \theta \ll 1$. Since for each local differential area the irradiance energy conserves, therefore there is a simple relationship between intensity $I(\mathbf{r}')$ at sensor plane and intensity $J(\mathbf{r})$ at mask plane:

$$I(\mathbf{r}')\,\mathrm{d}\mathbf{r}' = J(\mathbf{r})\,\mathrm{d}\mathbf{r}. \tag{S.58}$$

Differentiate Eq. (S.57), we get:

$$\mathrm{d}\mathbf{r}' = \left(1 + \frac{\lambda z}{2\pi}\nabla^2\phi(\mathbf{r})\right)\mathrm{d}\mathbf{r}. \tag{S.59}$$

Given Eq. (S.59), Eq. (S.58) can be reformulated as:

$$I(\mathbf{r}') = \frac{1}{\left(1 + \frac{\lambda z}{2\pi}\nabla^2\phi(\mathbf{r})\right)} J(\mathbf{r}) \approx \left(1 - \frac{\lambda z}{2\pi}\nabla^2\phi(\mathbf{r})\right) J(\mathbf{r}), \tag{S.60}$$

where the approximation is valid because $\frac{\lambda z}{2\pi}\nabla^2\phi(\mathbf{r}) \ll 1$. By Eq. (S.57), finally we arrive at:

$$I\left(\mathbf{r} + \frac{\lambda z}{2\pi}\nabla\phi\right) = \left(1 - \frac{\lambda z}{2\pi}\nabla^2\phi\right) J(\mathbf{r}). \tag{S.61}$$

References

1. Morgan, K. S., Paganin, D. M. & Siu, K. K. X-ray phase imaging with a paper analyzer. *Appl. Phys. Lett.* **100**, 124102 (2012).
2. Bérujon, S., Ziegler, E., Cerbino, R. & Peverini, L. Two-dimensional x-ray beam phase sensing. *Phys. Rev. Lett.* **108**, 158102 (2012).
3. Bérujon, S., Wang, H. & Sawhney, K. X-ray multimodal imaging using a random-phase object. *Phys. Rev. A* **86**, 063813 (2012).
4. Bérujon, S., Wang, H., Pape, I. & Sawhney, K. X-ray phase microscopy using the speckle tracking technique. *Appl. Phys. Lett.* **102**, 154105 (2013).
5. Zanette, I. *et al.* Speckle-based x-ray phase-contrast and dark-field imaging with a laboratory source. *Phys. review letters* **112**, 253903 (2014).
6. Bérujon, S. & Ziegler, E. Near-field speckle-scanning-based x-ray imaging. *Phys. Rev. A* **92**, 013837 (2015).

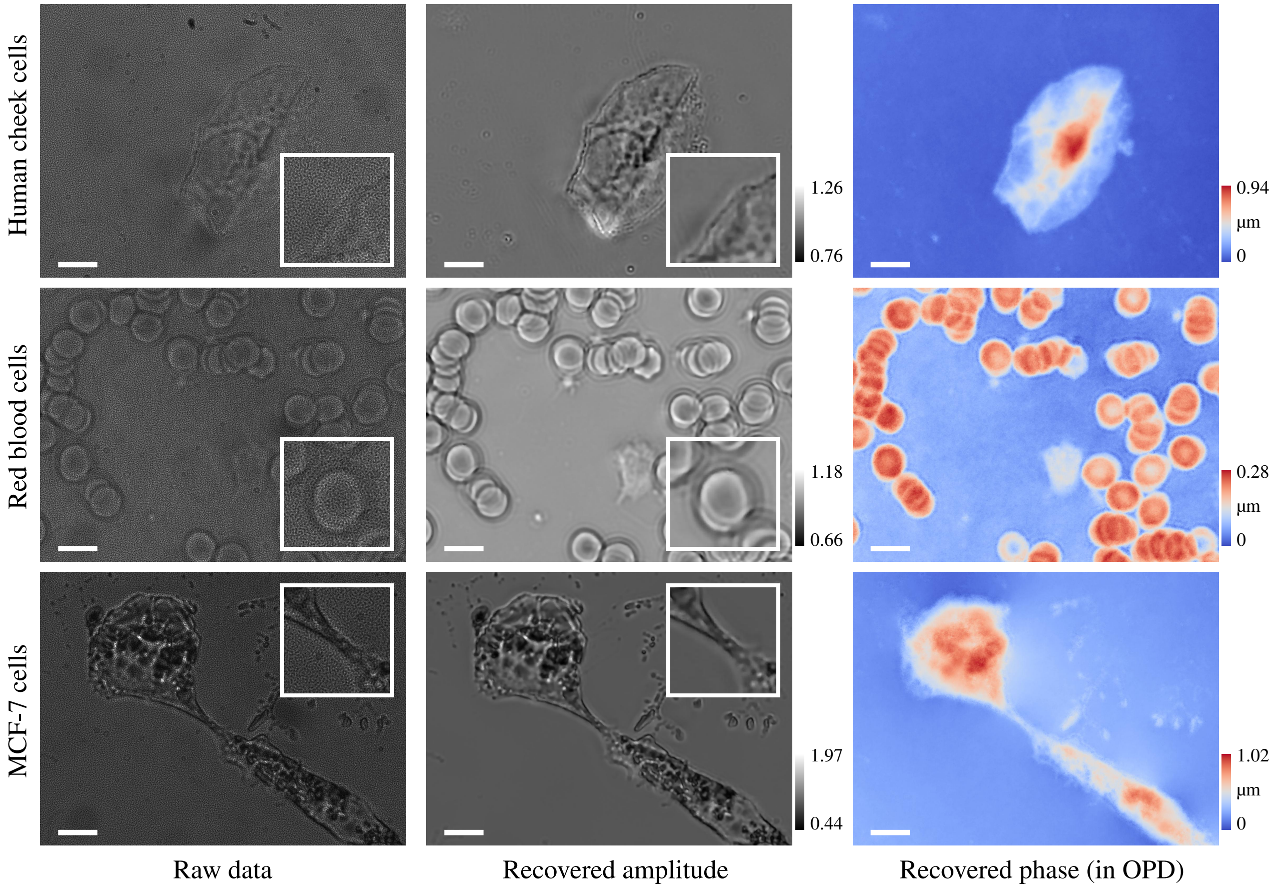

Figure S.6. Additional experimental results with unstained thin transparent cells with the proposed quantitative phase imaging pipeline. Images were taken under a $\times 100$ Mitutoyo plan apochromat objective, 0.70 NA. Inset close-up images show local areas of interest where in the recovered amplitude images the speckle patterns have been fully removed. Scale bars are 10 µm.

7. Wang, C., Dun, X., Fu, Q. & Heidrich, W. Ultra-high resolution coded wavefront sensor. *Opt. Express* **25**, 13736–13746 (2017).

8. Berto, P., Rigneault, H. & Guillon, M. Wavefront sensing with a thin diffuser. *Opt. Lett.* **42**, 5117–5120 (2017).

9. Lu, L. *et al.* Quantitative phase imaging camera with a weak diffuser based on the transport of intensity equation. In *Computational Imaging IV*, vol. 10990, 1099009 (International Society for Optics and Photonics, 2019).

10. Teague, M. R. Deterministic phase retrieval: a green's function solution. *JOSA A* **73**, 1434–1441 (1983).

11. Goodman, J. W. *Introduction to Fourier optics* (Roberts and Company Publishers, 2005).

12. Damberg, G. & Heidrich, W. Efficient freeform lens optimization for computational caustic displays. *Opt. express* **23**, 10224–10232 (2015).

13. Roddier, F. Curvature sensing and compensation: a new concept in adaptive optics. *Appl. Opt.* **27**, 1223–1225 (1988).

14. Roddier, F. Wavefront sensing and the irradiance transport equation. *Appl. optics* **29**, 1402–1403 (1990).

15. Gureyev, T., Roberts, A. & Nugent, K. Partially coherent fields, the transport-of-intensity equation, and phase uniqueness. *JOSA A* **12**, 1942–1946 (1995).

16. Gureyev, T. E. & Nugent, K. A. Rapid quantitative phase imaging using the transport of intensity equation. *Opt. communications* **133**, 339–346 (1997).

17. Guigay, J. P., Langer, M., Boistel, R. & Cloetens, P. Mixed transfer function and transport of intensity approach for phase retrieval in the fresnel region. *Opt. letters* **32**, 1617–1619 (2007).

18. Waller, L., Tian, L. & Barbastathis, G. Transport of intensity phase-amplitude imaging with higher order intensity derivatives. *Opt. Express* **18**, 12552–12561 (2010).

19. Kou, S. S., Waller, L., Barbastathis, G. & Sheppard, C. J. Transport-of-intensity approach to differential interference contrast (ti-dic) microscopy for quantitative phase imaging. *Opt. letters* **35**, 447–449 (2010).

20. Zuo, C., Chen, Q., Qu, W. & Asundi, A. High-speed transport-of-intensity phase microscopy with an electrically tunable lens. *Opt. express* **21**, 24060–24075 (2013).

21. Zuo, C., Chen, Q., Qu, W. & Asundi, A. Noninterferometric single-shot quantitative phase microscopy. *Opt. letters* **38**, 3538–3541 (2013).

22. Morgan, K. S., Paganin, D. M. & Siu, K. K. Quantitative single-exposure x-ray phase contrast imaging using a single attenuation grid. *Opt. express* **19**, 19781–19789 (2011).

23. Morgan, K. S. *et al.* A sensitive x-ray phase contrast technique for rapid imaging using a single phase grid analyzer. *Opt. letters* **38**, 4605–4608 (2013).

24. Chanteloup, J.-C. Multiple-wave lateral shearing interferometry for wave-front sensing. *Appl. optics* **44**, 1559–1571 (2005).

25. Bon, P., Maucort, G., Wattellier, B. & Monneret, S. Quadriwave lateral shearing interferometry for quantitative phase microscopy of living cells. *Opt. express* **17**, 13080–13094 (2009).

26. Wang, H. *et al.* X-ray wavefront characterization using a rotating shearing interferometer technique. *Opt. express* **19**, 16550–16559 (2011).

27. Wen, H. H., Bennett, E. E., Kopace, R., Stein, A. F. & Pai, V. Single-shot x-ray differential phase-contrast and diffraction imaging using two-dimensional transmission gratings. *Opt. letters* **35**, 1932–1934 (2010).

28. Horn, B. K. & Schunck, B. G. Determining optical flow. *Artif. intelligence* **17**, 185–203 (1981).

29. Lucas, B. D. & Kanade, T. An iterative image registration technique with an application to stereo vision. In *Proceedings of Imaging Understanding Workshop*, 121–130 (Vancouver, British Columbia, 1981).

30. Roddier, C. & Roddier, F. Interferogram analysis using fourier transform techniques. *Appl. optics* **26**, 1668–1673 (1987).

31. Takeda, M. Fourier fringe analysis and its application to metrology of extreme physical phenomena: a review. *Appl. optics* **52**, 20–29 (2013).

32. Ihrke, I., Wetzstein, G. & Heidrich, W. A theory of plenoptic multiplexing. In *Computer Vision and Pattern Recognition (CVPR), 2010 IEEE Conference on*, 483–490 (IEEE, 2010).

33. Rudin, L. I., Osher, S. & Fatemi, E. Nonlinear total variation based noise removal algorithms. *Phys. D: nonlinear phenomena* **60**, 259–268 (1992).

34. Boyd, S., Parikh, N., Chu, E., Peleato, B. & Eckstein, J. Distributed optimization and statistical learning via the alternating direction method of multipliers. *Foundations Trends Mach. Learn.* **3**, 1–122 (2011).

35. Parikh, N. & Boyd, S. Proximal algorithms. *Foundations Trends optimization* **1**, 127–239 (2014).

36. Bing, P., Hui-Min, X., Bo-Qin, X. & Fu-Long, D. Performance of sub-pixel registration algorithms in digital image correlation. *Meas. Sci. Technol.* **17**, 1615 (2006).

37. Almeida, M. S. & Figueiredo, M. Deconvolving images with unknown boundaries using the alternating direction method of multipliers. *IEEE Trans. Image Process.* **22**, 3074–3086 (2013).

38. Wang, C., Fu, Q., Dun, X. & Heidrich, W. Megapixel adaptive optics: towards correcting large-scale distortions in computational cameras. *ACM Transactions on Graph. (TOG)* **37**, 115 (2018).

39. Brox, T., Bruhn, A., Papenberg, N. & Weickert, J. High accuracy optical flow estimation based on a theory for warping. In *Proc. ECCV*, 25–36 (Springer, 2004).

40. Matsushima, K. & Shimobaba, T. Band-limited angular spectrum method for numerical simulation of free-space propagation in far and near fields. *Opt. Express* **17**, 19662–19673 (2009).
